# Epigenetic Profiling of Cell-Free DNA in Cerebrospinal Fluid: A Novel Biomarker Approach for Metabolic Brain Diseases

**DOI:** 10.3390/life15081181

**Published:** 2025-07-25

**Authors:** Kyle Sporn, Rahul Kumar, Kiran Marla, Puja Ravi, Swapna Vaja, Phani Paladugu, Nasif Zaman, Alireza Tavakkoli

**Affiliations:** 1 Department of Medicine, Norton College of Medicine, SUNY Upstate Medical University, 785 E Adams St., Syracuse, NY 13202, USA; 2 Department of Biochemistry and Molecular Biology, University of Miami Miller School of Medicine, Miami, FL 33136, USA; rxk641@miami.edu; 3 Carver College of Medicine, University of Iowa, 375 Newton Rd., Iowa City, IA 52242, USA; kmarla@uiowa.edu; 4 Department of Biology, University of Michigan, 500 S State St., Ann Arbor, MI 48109, USA; puja.mi.us@gmail.com; 5 Rush Medical College, 600 S Paulina St. Suite 524, Chicago, IL 60612, USA; swapna_vaja@rush.edu; 6 Sidney Kimmel Medical College, Thomas Jefferson University, 1025 Walnut St. #100, Philadelphia, PA 19107, USA; phani.paladugu@students.jefferson.edu; 7 Human–Machine Perception Laboratory, Department of Computer Science, University of Nevada, 1664 N. Virginia St., Reno, NV 89557, USA; zaman@nevada.unr.edu (N.Z.); tavakkol@unr.edu (A.T.)

**Keywords:** epigenetic profiling, metabolic brain diseases, cerebrospinal fluid, MELAS, cfMeDIP-seq

## Abstract

Due to their clinical heterogeneity, nonspecific symptoms, and the limitations of existing biomarkers and imaging modalities, metabolic brain diseases (MBDs), such as mitochondrial encephalopathies, lysosomal storage disorders, and glucose metabolism syndromes, pose significant diagnostic challenges. This review examines the growing potential of cell-free DNA (cfDNA) derived from cerebrospinal fluid (CSF) epigenetic profiling as a dynamic, cell-type-specific, minimally invasive biomarker approach for MBD diagnosis and monitoring. We review important technological platforms and their use in identifying CNS-specific DNA methylation patterns indicative of neuronal injury, neuroinflammation, and metabolic reprogramming, including cfMeDIP-seq, enzymatic methyl sequencing (EM-seq), and targeted bisulfite sequencing. By synthesizing current findings across disorders such as MELAS, Niemann–Pick disease, Gaucher disease, GLUT1 deficiency syndrome, and diabetes-associated cognitive decline, we highlight the superior diagnostic and prognostic resolution offered by CSF cfDNA methylation signatures relative to conventional CSF markers or neuroimaging. We also address technical limitations, interpretive challenges, and translational barriers to clinical implementation. Ultimately, this review explores CSF cfDNA epigenetic analysis as a liquid biopsy modality. The central objective is to assess whether epigenetic profiling of CSF-derived cfDNA can serve as a reliable and clinically actionable biomarker for improving the diagnosis and longitudinal monitoring of metabolic brain diseases.

## 1. Introduction

A broad and frequently debilitating category of inherited and acquired illnesses are metabolic brain diseases (MBDs), also referred to as neurometabolic disorders. Disruptions in vital cellular functions, such as energy production, substrate metabolism, or detoxification pathways, are what essentially define these disorders [1]. A variety of severe neurological symptoms, including cognitive decline, motor disturbances, and seizures, are often the result of such metabolic dysfunctions, which often lead to progressive neurodegeneration and significantly reduce patients’ quality of life and lifespan [1]. Clinicians face significant diagnostic challenges due to the inherent heterogeneity of these disorders and their variable age of onset, which can range from neonatal to adulthood [1,2].

While traditionally focused on rare inherited conditions such as mitochondrial or lysosomal storage disorders, the scope of MBDs is increasingly understood to include acquired metabolic insults to the brain. This broader categorization reflects mounting evidence that chronic systemic metabolic dysfunction—such as in diabetes mellitus—can directly contribute to CNS pathology and cognitive decline via impaired glucose transport, oxidative stress, and altered energy metabolism. For this reason, conditions like diabetes-associated cognitive decline (DCD) are considered within the metabolic brain disease continuum in this review, even though they are not classically categorized as inborn errors of metabolism.

The need for less invasive but highly informative methods has led to a significant evolution in the diagnostic landscape for diseases of the central nervous system (CNS). In the past, invasive techniques like lumbar punctures and brain biopsies have been necessary for the conclusive diagnosis of numerous CNS disorders. These techniques offer direct tissue access, but they come with risks, such as infection and bleeding, and they might not adequately represent the molecular diversity that defines a number of neurological conditions [3]. Liquid biopsy has become a revolutionary substitute in this regard. By examining circulating biomarkers present in different body fluids, this minimally invasive method provides a flexible and approachable way to detect, track, and characterize diseases at the molecular level [3]. Among the various analytes in liquid biopsy, cell-free DNA (cfDNA) has garnered considerable attention. Released from dying cells into the circulation, cfDNA provides a “real-time” molecular snapshot of ongoing pathological processes within the body [4].

**Figure 1:**
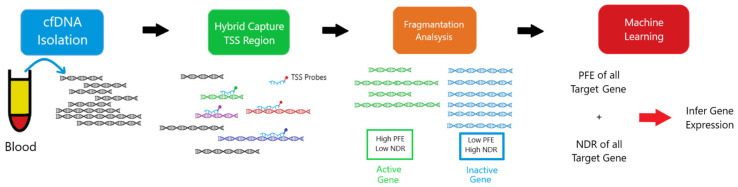
This flowchart illustrates the EPIC-Seq workflow for analyzing cell-free DNA (cfDNA) from liquid biopsies. Targeted sequencing near gene transcription start sites enables extraction of fragmentomic features like promoter fragmentation entropy and nucleosome-depleted region depth. These features train a machine learning model to infer gene expression for diagnostic and prognostic use. This file is made available under the Creative Commons CC0 1.0 Universal Public Domain Dedication with permission from Wikimedia Commons [20].

While the utility of cfDNA in liquid biopsy has been extensively explored in oncology, its potential in non-malignant neurological disorders, particularly MBDs, is now gaining significant momentum [5,6,7,8,9]. A compelling aspect of cfDNA analysis lies in the interrogation of epigenetic modifications, most notably DNA methylation patterns. These epigenetic marks are stable, inherently tissue-specific, and critically reflect the gene regulatory changes that define cellular identity and function [9,10,11,12]. Crucially, the unique epigenetic signatures carried by cfDNA fragments can reveal their tissue and even cell type of origin, enabling the precise mapping of CNS-specific damage or dysfunction, with cerebrospinal fluid (CSF)—which directly bathes the brain and spinal cord—standing out as an ideal biofluid for capturing CNS-derived cfDNA (Figure 1) [9,10,11,12,13,14,15,16,17,18,19,20]. This direct anatomical proximity allows CSF to overcome the limitations imposed by the blood–brain barrier (BBB), which often restricts the passage of CNS-derived biomarkers into the peripheral bloodstream, thereby limiting the utility of plasma-based analyses for many brain pathologies [3]. This review aims to critically examine the emerging role of epigenetic profiling of CSF-derived cfDNA, exploring its potential to revolutionize the diagnostic and prognostic landscape for MBDs by providing unprecedented molecular-level insights into CNS metabolic dysfunction.

## 2. Methodology

This narrative review was conducted to synthesize current knowledge regarding the diagnostic and prognostic utility of cerebrospinal fluid (CSF)-derived cell-free DNA (cfDNA) methylation profiling in metabolic brain diseases (MBDs). A comprehensive literature search was conducted across the PubMed, Scopus, and Web of Science databases from their inception through June 2025. Search terms included combinations of “cerebrospinal fluid”, “cfDNA”, “cell-free DNA”, “DNA methylation”, “epigenetics”, “liquid biopsy”, “metabolic brain disease”, “mitochondrial encephalopathy”, “lysosomal storage disease”, “GLUT1 deficiency”, and “diabetes-associated cognitive decline”. Only articles published in English were considered.

Eligible sources included peer-reviewed primary research articles, systematic reviews, epigenetic technology reports, and consensus statements addressing either traditional or emerging CSF biomarkers in neurologic and neurometabolic conditions. Studies were included if they described or evaluated CSF-based biomarkers in metabolic brain diseases, investigated cfDNA methylation or epigenetic alterations relevant to central nervous system pathology, or offered mechanistic or translational insights with potential clinical implications. Case reports lacking biomarker analysis, studies not involving CSF or cfDNA, and non-CNS-focused investigations were excluded. Abstract-only conference proceedings without accessible full-text data were also omitted.

In instances where multiple studies addressed similar topics, such as cfDNA profiling in MELAS or lysosomal storage disorders, priority was given to recent, high-impact, or methodologically rigorous publications. Technical platforms—including cfMeDIP-seq, enzymatic methyl-sequencing (EM-seq), and bisulfite-based approaches—were reviewed using both primary validation studies and, where necessary, technical documentation from established protocols.

The synthesis of findings was organized thematically around four key domains: (1) traditional CSF biomarkers used in metabolic brain disease diagnosis and monitoring; (2) the emerging methodologies for cfDNA methylation profiling, including technological innovations and limitations; (3) disease-specific applications and insights across select disorders such as MELAS, Niemann-Pick, and GLUT1 deficiency syndrome; and (4) barriers to clinical translation, including technical challenges, data interpretation, regulatory considerations, and ethical concerns. This thematic approach enabled a structured, integrative perspective on how cfDNA methylation profiling may complement or surpass conventional diagnostic modalities in the evaluation of metabolic brain disorders.

## 3. Metabolic Brain Diseases: Clinical Spectrum and Diagnostic Imperatives

The diagnosis of neurometabolic disorders presents a significant challenge due to their inherent heterogeneity and the often nonspecific nature of their clinical presentations. This section delineates the diverse manifestations and current diagnostic limitations of key metabolic brain diseases, underscoring the pressing need for advanced biomarker strategies.

### 3.1. Mitochondrial Encephalopathies (e.g., MELAS)

**Figure 2:**
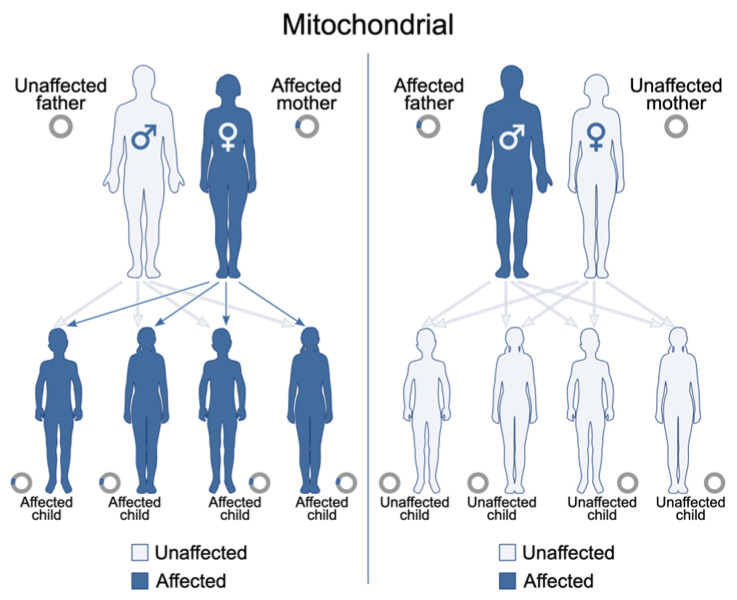
Diagram showing mitochondrial inheritance. This file is licensed under the Creative Commons Attribution-Share Alike 4.0 International license with permission from Wikimedia Commons [22].

**Figure 3:**
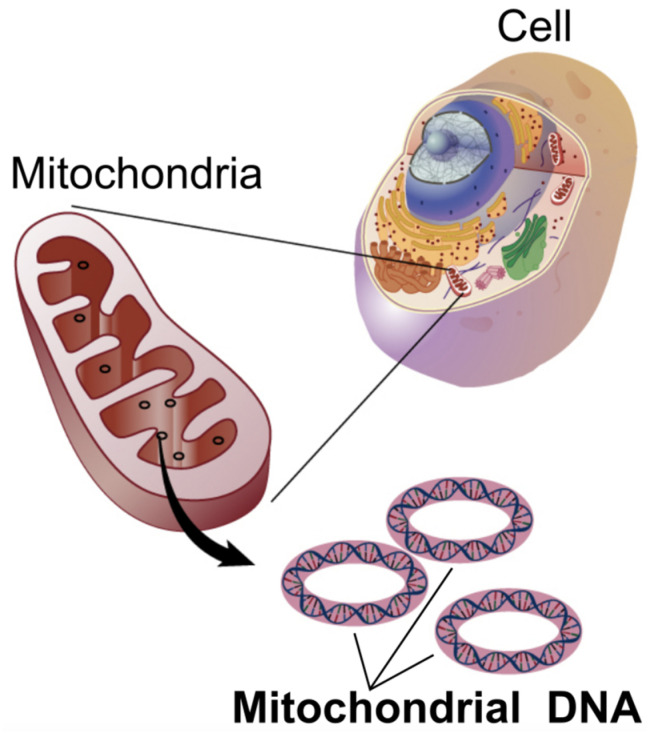
Mitochondrial DNA is a small, circular chromosome located within the mitochondria, organelles responsible for producing energy in cells. Because mitochondria are inherited maternally, mitochondrial DNA is passed from mothers to their offspring. This image is a work of the National Institutes of Health, part of the United States Department of Health and Human Services, taken or made as part of an employee’s official duties. As a work of the U.S. federal government, the image is in the public domain. Permission was granted by Wikimedia Commons [23].

Mitochondrial encephalomyopathy, lactic acidosis, and stroke-like episodes (MELAS) is a maternally inherited mitochondrial disorder primarily caused by mutations in mitochondrial DNA (mtDNA), with the m.3243A>G mutation in the *MT-TL1* gene being the most common genetic culprit (Figure 2 and Figure 3 and Table 1) [21,22,23]. These genetic alterations compromise the mitochondria’s ability to synthesize proteins, utilize oxygen, and generate energy, leading to widespread cellular dysfunction, particularly in metabolically demanding tissues such as the brain and muscles [21,24,25].

Clinically, MELAS typically manifests in childhood, often between 2 and 10 years of age, following a period of seemingly normal early psychomotor development [21,24,25]. A hallmark feature of the syndrome is the occurrence of stroke-like episodes, which frequently appear before the age of 40. These episodes can present with varied neurological deficits, including aphasia, cortical vision loss, motor weakness (hemiparesis), severe headaches, seizures, and altered mental status, leading to a progressive accumulation of neurological impairment over time [21,24,25]. Other common symptoms include encephalopathy (manifesting as seizures and/or dementia), recurrent migrainous headaches often accompanied by vomiting, generalized muscle weakness (myopathy), exercise intolerance, and chronic lactic acidosis detectable in both blood and CSF [21,24,25]. Patients may also exhibit short stature and progressive sensorineural hearing loss [21,24,25]. Less frequently observed manifestations include involuntary muscle spasms (myoclonus), impaired muscle coordination (ataxia), cardiac and renal complications, diabetes mellitus, and various psychiatric disturbances [21,24,25].

**Figure 4:**
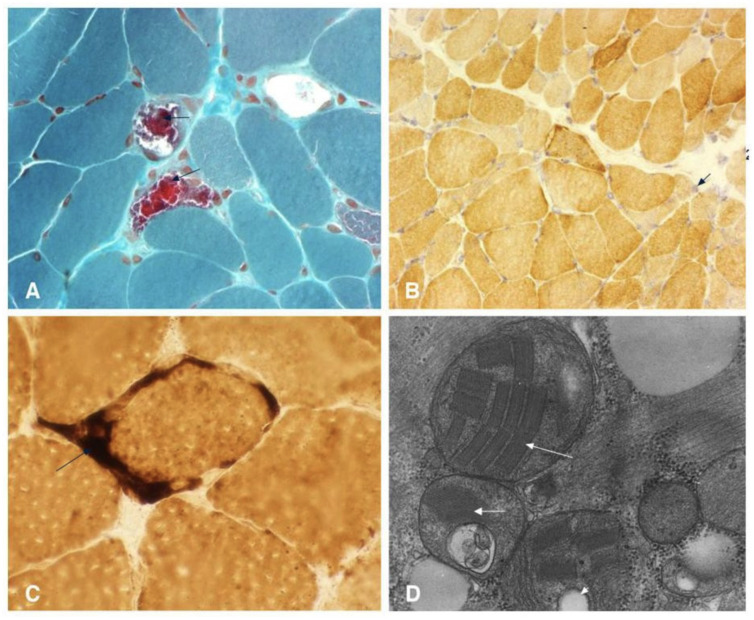
(**A**) Modified Gomori trichrome staining reveals multiple ragged red fibers (arrowhead). (**B**) Cytochrome c oxidase staining demonstrates lightly stained Type I fibers, darker Type II fibers, and several fibers containing abnormal mitochondrial aggregates (arrowhead). Note the presence of cytochrome c oxidase–negative fibers, a characteristic finding in mitochondrial encephalopathy, lactic acidosis, and stroke-like episodes (MELAS). (**C**) Succinate dehydrogenase staining highlights several ragged blue fibers, along with intense mitochondrial staining in vascular structures (arrow). (**D**) Electron microscopy shows abnormal mitochondrial accumulation featuring paracrystalline inclusions (arrowhead), osmiophilic inclusions (large arrowhead), and mitochondrial vacuolization (small arrowhead). This file is licensed under the Creative Commons Attribution 2.0 Generic license with permission granted by Wikimedia Commons [26].

Despite these characteristic features, current diagnostic approaches for MELAS face several limitations. While elevated lactate levels in blood and CSF are common, this finding is not specific to MELAS and can be observed in a range of other mitochondrial diseases, metabolic disorders, or systemic illnesses [21,24,25]. Notably, a minority of individuals with MELAS may even present with normal serum lactic acid levels, further complicating diagnosis [21,24,25]. The genetic diagnosis of MELAS is complicated by mitochondrial heteroplasmy, which refers to the co-existence of mutated and normal mtDNA within the same individual. The clinical expression of MELAS is highly dependent on the proportion and tissue distribution of the mutated mtDNA [21,24,25]. This phenomenon means that the pathogenic mtDNA variant may be undetectable in readily accessible samples, such as leukocytes, often necessitating genetic testing from other tissues, including buccal mucosa, urinary sediment, or skeletal muscle [21,24,25]. This variability in mutational load across tissues can lead to false-negative results if only blood samples are analyzed [21,24,25]. Furthermore, while a muscle biopsy revealing ragged red fibers (RRFs) is suggestive of mitochondrial myopathy, it is not always a mandatory or definitive diagnostic criterion, as biochemical results from muscle tissue can sometimes appear normal despite the presence of MELAS (Figure 4 and Table 1) [21,24,25]. Neuroimaging, such as MRI, may reveal stroke-like lesions that do not conform to typical vascular territories; however, these findings are also not unique to MELAS and can be observed in other conditions [21,24,25].

The non-specific and variable nature of MELAS symptoms, coupled with the complexities of heteroplasmy in traditional genetic testing, frequently leads to underdiagnosis or significant delays in establishing a definitive diagnosis. The current diagnostic reliance on invasive procedures like muscle biopsy or spinal tap, or on tests limited by tissue-specific mutational distribution, means that patients often endure a prolonged diagnostic odyssey. This “hidden” disease burden suggests that the true prevalence of MELAS may be higher than current estimates, which range from 1 in 500,000 to 16 in 100,000 in studies from Japan and Finland, respectively [21,24,25]. This highlights a critical need for more sensitive, less invasive, and comprehensive diagnostic tools capable of overcoming the challenges posed by tissue-specific heteroplasmy and non-specific clinical presentations, for which CSF cfDNA epigenetic profiling offers a promising avenue.

**Table 1:** Clinical manifestations and diagnostic features of key metabolic brain diseases.

Disease Category	Example Diseases	Key Clinical Manifestations	Common Diagnostic Methods	Key Diagnostic Limitations
Mitochondrial Encephalopathies [21,24,25,27,28]	MELAS	Stroke-like episodes (pre-40), encephalopathy (seizures/dementia), lactic acidosis (blood/CSF), myopathy, recurrent headaches, short stature, hearing impairment.	Increased Blood/CSF lactate, Increased lactate: pyruvate, muscle biopsy (ragged red fibers), mtDNA genetic testing.	Non-specific lactic acidosis, heteroplasmy/tissue distribution challenges for genetic testing, muscle biopsy not always definitive, neuroimaging non-specific.
Lysosomal Storage Diseases [29,30,31,32,33]	Niemann-Pick (Types A, B, C), Gaucher (Types 1, 2, 3)	Early onset: Hepatosplenomegaly, developmental delay, hypotonia, jaundice. Later onset: Progressive neurodegeneration (ataxia, dementia, seizures), vertical gaze palsy (NPC), organomegaly, musculoskeletal issues, psychiatric symptoms (NPC).	Biochemical enzyme assays, mutational analysis (e.g., *SMPD1*, *NPC1*, *NPC2*, *GBA1* genes), oxysterols (NPC).	High index of suspicion needed, biomarker non-specificity (oxysterols), sample stability issues, genetic testing complexities (polymorphism, VUS), non-specific neuroimaging, clinical assessment difficulties.
Glucose Metabolism Disorders [34,35,36,37,38]	GLUT1 Deficiency Syndrome, Diabetes-Associated Cognitive Decline	GLUT1DS: Early-onset seizures, developmental delay, cognitive impairment, movement disorders, microcephaly, speech/language issues. DCD: Attention/memory/executive function deficits, visuospatial decline, increased dementia risk.	GLUT1DS: CSF glucose/lactate, *SLC2A1* genetic testing. DCD: Neurocognitive assessment, blood glucose/HbA1c.	GLUT1DS: Symptom variability/underdiagnosis, CSF glucose/lactate can be near normal, genetic mimics (*PURA*, *HK1*), functional assay availability. DCD: Non-specific cognitive symptoms, early metabolic changes precede symptoms, overlap/mimicry with AD/other dementias, complex pathophysiology.

### 3.2. Lysosomal Storage Diseases (e.g., Niemann–Pick, Gaucher)

Lysosomal storage diseases (LSDs) are a broad category of over 40 inherited metabolic disorders, each stemming from a deficiency in one of the lysosomal enzymes. This enzymatic defect leads to the progressive accumulation of undegraded substrates within lysosomes across various organs and tissues, ultimately causing cellular dysfunction and damage [29,30].

**Figure 5:**
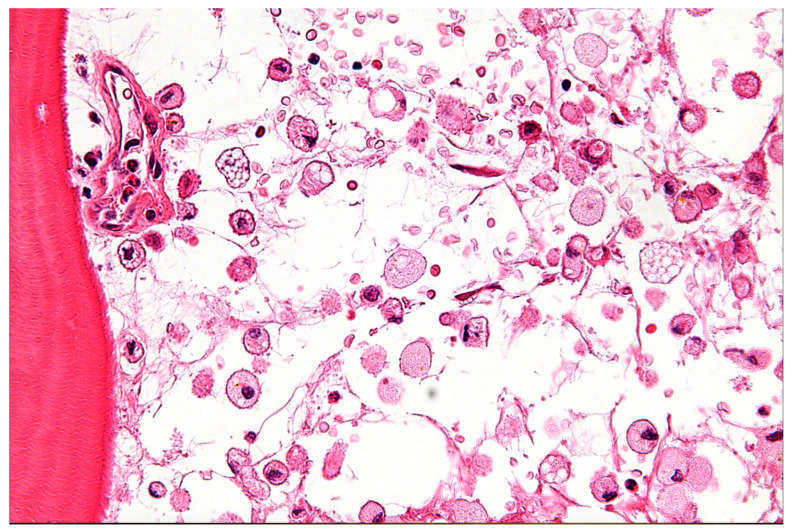
High-power micrograph of Gaucher disease with associated necrotic bone, stained with hematoxylin and eosin (H&E). Histologically, necrotic bone is identified by the absence of osteocytes within the lacunae. This file is licensed under the Creative Commons Attribution-Share Alike 3.0 Unported license with permission from Wikimedia Commons [39].

Niemann–Pick Disease (NPD) and Gaucher Disease (GD) are two of the most well-known LSDs. NPD mainly affects how the body uses and breaks down fats, including cholesterol and other lipids, inside cells, which causes them to build up toxically and eventually cause cell death [30,31,32,33]. NPD Types A and B are specifically brought on by mutations in the SMPD1 gene, which lead to a sphingomyelinase enzyme deficiency. On the other hand, NPD Type C results from intracellular cholesterol transport-related mutations in the NPC1 or NPC2 genes [30,31,32,33]. Due to mutations in the GBA1 gene, Gaucher Disease (GD) is characterized by a lack of the lysosomal enzyme glucocerebrosidase. This deficiency leads to the accumulation of glucosylceramide within macrophages, forming characteristic “Gaucher cells” that infiltrate various organs (Figure 5 and Table 1) [31,32].

The clinical manifestations of LSDs are remarkably diverse and highly dependent on the specific enzyme deficiency and the patient’s age of onset [29,30,31,32,33,39]. In early childhood, severe forms like NPD Type A often present with significant visceral involvement, including hepatosplenomegaly, prolonged jaundice, and pulmonary infiltrates, alongside neurological signs such as hypotonia, developmental delay, and failure to thrive [29,30,31,32,33,39]. As patients progress into later childhood or adolescence, conditions like NPD Type C and GD Type 3 are often dominated by progressive neurodegeneration, manifesting as ataxia, dysarthria, dysphagia, epileptic seizures, dystonia, and a progressive decline in cognitive function leading to dementia or intellectual disability [29,30,31,32,33,39]. Specific neurological signs like vertical supranuclear gaze palsy are highly characteristic of NPD Type C [29,30,31,32,33,39]. Other symptoms can include pain, organ enlargement, musculoskeletal abnormalities, muscle weakness, hearing loss, and corneal clouding [29,30,31,32,33,39]. In adults, particularly with NPD Type C or GD Type 1, presentations may be subtle, predominantly featuring early-onset dementia or psychiatric manifestations, though careful examination often reveals underlying neurological signs [33]. It is noteworthy that non-neuronopathic GD Type 1 can increase susceptibility to Parkinson’s disease later in life [29,30,31,32,33,39].

Despite the availability of biochemical tests and mutational analysis, which can generally confirm the diagnosis of LSDs [29,30,31,32,33,39], significant diagnostic limitations persist. A high index of suspicion is paramount for diagnosis due to the diverse and often nonspecific nature of early symptoms [33]. For Niemann-Pick Type C (NP-C), specific challenges abound. Biomarkers such as oxysterols (cholestane-3β,5α,6β-triol and 7-ketocholesterol) are elevated in NP-C but are not exclusive to it, also appearing in NP-A, NP-B, and acid lipase deficiency, necessitating cautious interpretation [29,30,31,32,33,39]. Sample stability is another concern, as prolonged storage at room temperature can lead to cholesterol autoxidation, potentially yielding false-positive oxysterol results [29,30,31,32,33,39].

Genetic testing, while definitive, is complicated by the highly polymorphic nature of the *NPC1* gene, making the interpretation of novel mutations challenging. Routine sequencing methods may fail to detect large deletions/duplications or deep intronic changes, and variants of unknown significance (VUS) often require laborious functional assays for clarification [29,30,31,32,33,39]. The filipin staining test, once considered the gold standard, is no longer a first-line diagnostic tool and is now primarily reserved for assessing the functional significance of new genetic variants or confirming diagnoses when genetic testing is inconclusive [29,30,31,32,33,39]. Neuroimaging findings, such as MRI or PET, are typically non-specific and may not be present in the early stages of the disease, thus their absence does not rule out NP-C [33]. Clinically, the non-specific nature of symptoms like ataxia can lead to NP-C patients being “hidden” within broader patient cohorts, and challenges in psychiatric practice, such as incomplete medical histories, further impede timely diagnosis [33]. While Gaucher disease diagnosis is confirmed by demonstrating glucocerebrosidase deficiency in leukocytes, the broad clinical spectrum, including the potential for later-onset neurological complications like Parkinsonism in Type 1, can still delay timely identification [31,32,33,39].

**Figure 6:**
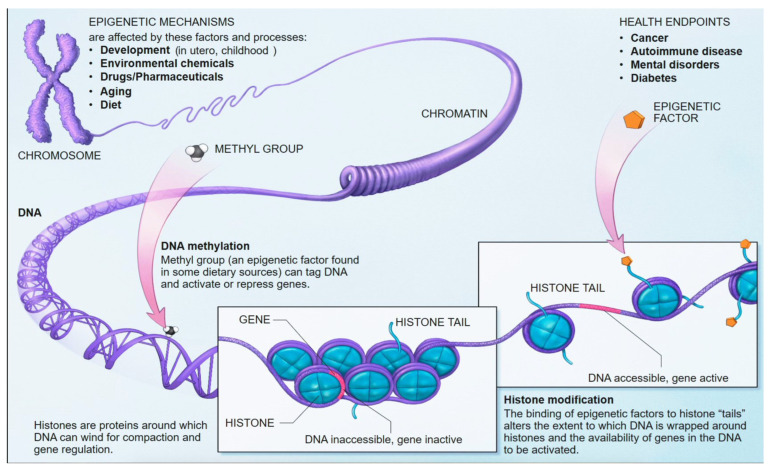
Epigenetic changes—shaped by factors like development, environment, aging, and diet—affect gene expression without altering DNA. DNA methylation and histone modifications regulate gene activity by changing DNA accessibility. These mechanisms can influence health and contribute to diseases like cancer, autoimmune disorders, mental illness, and diabetes. (Source: National Institutes of Health). This work is in the public domain in the United States because it is a work prepared by an officer or employee of the United States Government as part of that person’s official duties under the terms of Title 17, Chapter 1, Section 105 of the US Code with permission from Wikimedia Commons [40].

The profound heterogeneity of LSDs, encompassing a wide range of genetic causes and highly variable clinical presentations (including age of onset, organ involvement, and the balance between neurological and visceral symptoms), creates a complex diagnostic labyrinth. Even with established biochemical and genetic testing, the non-specific nature of early symptoms and the prevalence of variants of unknown significance can lead to substantial diagnostic delays and misdiagnoses, particularly for the neuronopathic forms. This complexity means that patients, especially those with atypical or adult-onset presentations, may experience prolonged diagnostic journeys, highlighting the inadequacy of current methods for early, definitive, and comprehensive diagnosis of CNS involvement. Epigenetic biomarkers, with their capacity to reflect tissue- and cell-specific changes and potentially capture subtle molecular alterations at an early stage, offer a promising approach to navigating this diagnostic complexity and identifying CNS involvement more promptly and accurately (Figure 6) [40].

### 3.3. Glucose Metabolism Disorders (e.g., GLUT1 Deficiency, Diabetes-Associated Cognitive Decline)

Disorders of glucose metabolism can profoundly impact brain function, ranging from rare genetic conditions to widespread acquired diseases. Two notable examples are Glucose Transporter Type 1 Deficiency Syndrome (GLUT1DS) and Diabetes-Associated Cognitive Decline (DCD).

GLUT1 Deficiency Syndrome (GLUT1DS): This is a rare genetic metabolic disorder caused by pathogenic variants in the *SLC2A1* gene, which encodes the GLUT1 protein [34]. A deficiency in GLUT1 impairs the crucial transport of glucose across the blood–brain barrier (BBB) into the brain, resulting in chronic neuroglycopenia—a state of insufficient glucose supply to the brain, which relies primarily on glucose for energy [34,35,36]. The clinical spectrum of GLUT1DS is highly variable [34,35,36]. The most common manifestation is epilepsy, with seizures often commencing within the first six months of life. These can include generalized tonic–clonic, myoclonic, atypical absence, and atonic seizures [34,35,36]. Beyond seizures, patients frequently exhibit deceleration of head growth during infancy, potentially leading to acquired microcephaly. Developmental delays and cognitive impairment, ranging from mild learning difficulties to severe intellectual disability, are also common [34,35,36]. Movement disorders are prevalent, such as diminished muscle tone (hypotonia), poor balance and coordination (ataxia), slow and stiff limb movements (spasticity), and awkward postures (dystonia), including paroxysmal exercise-induced dyskinesia (PED) [34,35,36]. Speech and language abnormalities, such as dysarthria and dysfluency, are also frequently observed [34,35,36]. Interestingly, some individuals may present with non-classic forms of GLUT1DS, experiencing movement disorders and cognitive impairment without epilepsy, or may even be asymptomatic due to mosaicism, often identified when symptomatic family members undergo genetic testing [34,35,36].

Diabetes-Associated Cognitive Decline (DCD): Cognitive impairment is a recognized and prevalent complication of both type 1 and type 2 diabetes [41]. The pathophysiology of DCD is complex and multifactorial, involving direct effects of chronic hyperglycemia, insulin dysregulation and resistance, microvascular and macrovascular pathologies, oxidative stress, chronic low-grade inflammation, mitochondrial dysfunction, neurotransmitter dysregulation, and even epigenetic modifications [41,42,43,44]. Clinically, DCD manifests as impairments across multiple cognitive domains, including attention and concentration, memory (both short-term/working and long-term), executive functions (e.g., planning, decision-making, inhibition), and visuospatial abilities [41,42,43,44,45,46]. These deficits can range from mild cognitive impairment (MCI) to overt dementia [46]. Diabetes significantly increases the risk of developing all types of dementia, including Alzheimer’s disease and vascular dementia, with some studies suggesting a 65% higher risk of Alzheimer’s in diabetics [46,47,48].

Current diagnostic approaches for these disorders of glucose metabolism face notable limitations. For GLUT1DS, despite the classic presentation, the wide variability in symptoms and severity means that mild cases are often undiagnosed, contributing to a significant gap between estimated true prevalence (at least 1:24,000) and diagnosed cases [34,35,36]. While reduced CSF glucose and lactate levels are key biochemical indicators, these levels can be near or within normal reference ranges, especially in milder presentations, making biochemical diagnosis challenging [34,35,36,41,42,43,44,45,46]. Genetic testing for *SLC2A1* gene variants remains the gold standard for confirmation; however, variants in other genes (e.g., *PURA*, *HK1*) can present with similar CSF biomarker profiles, complicating differential diagnosis. For instance, *HK1* variants result in low CSF glucose but *abnormally high* CSF lactate, which distinguishes them from GLUT1DS, where lactate is low-normal or low [44,45,46,47,48]. Functional assays, such as erythrocyte GLUT1 activity or protein quantification, are available but not universally accessible or definitive [44,45,46,47,48]. Neuroimaging, including PET scans, can show reduced brain metabolism, but their accuracy and reliability in establishing a definitive diagnosis are not yet fully established [44,45,46,47,48].

For DCD, the cognitive symptoms (e.g., memory, language, judgment issues) are often non-specific and can overlap considerably with normal age-related cognitive changes or other neurodegenerative conditions like Alzheimer’s disease [46,47,48]. Given that Type 2 Diabetes is known to hasten brain aging and cognitive decline, much like early Alzheimer’s pathology, this mimicry is especially difficult [44]. Additionally, changes in cerebral glucose metabolism, a major contributing factor to DCD, can occur decades before overt cognitive dysfunction manifests, making early detection challenging with available clinical tools [43]. It is difficult to identify precise, actionable diagnostic markers or intervention targets due to the intricate and multifactorial pathophysiology of DCD, which involves a network of interrelated mechanisms (hyperglycemia, insulin resistance, vascular damage, and neuroinflammation) [41].

The “silent progression” pattern shared by GLUT1DS and DCD is typified by early, subtle symptoms that are frequently missed or misdiagnosed. DCD specifically highlights how CNS pathology can be fueled by systemic metabolic dysfunction, like diabetes, through intricately linked mechanisms that closely resemble those of other neurodegenerative diseases. The crucial need for biomarkers that can identify disease processes before overt clinical symptoms appear or that can precisely distinguish these conditions from other neurodegenerative disorders or common age-related changes is highlighted by this clinical mimicry and the frequently subclinical onset. Present-day biomarkers are frequently non-specific or only show up when the disease is advanced. Epigenetic profiling of CSF cfDNA could offer a unique advantage by capturing early, subtle molecular shifts related to brain cell dysfunction or metabolic reprogramming, potentially enabling earlier and more accurate differentiation of these conditions, and distinguishing them from other conditions with similar symptoms (Table 1) [41,42,43,44,45,46,47,48].

### 3.4. Overarching Challenges in the Diagnosis of Neurometabolic Disorders

The collective experience with MELAS, LSDs, and glucose metabolism disorders reveals several overarching challenges in the diagnosis of neurometabolic conditions. These diseases are individually rare but collectively numerous and highly heterogeneous, presenting with complex clinical pictures that can vary dramatically between pediatric and adult presentations [1,2]. This phenotypic complexity often leads to significant diagnostic delays and underdiagnosis, as symptoms frequently mimic more common neurological disorders [1,2]. Many symptoms, such as developmental delay, cognitive impairment, seizures, and movement disorders, are nonspecific and overlap across various MBDs and other neurological conditions, making differential diagnosis difficult [1,2]. While biochemical and genetic tests are often definitive for many MBDs, some still necessitate invasive procedures like muscle biopsies or spinal taps, which carry inherent risks and patient discomfort [25]. Furthermore, traditional diagnostic measures often lack the sensitivity required for early disease detection, frequently revealing pathology only in late phases when potential therapeutic windows may have already closed [15]. These collective limitations underscore the urgent need for novel, non-invasive, and highly sensitive diagnostic tools that can provide early, specific, and comprehensive molecular insights into CNS metabolic dysfunction (Table 1).

Table 1: This table serves as a crucial summary, consolidating complex information about the diverse clinical presentations and, more importantly, the shared and specific diagnostic limitations across different categories of metabolic brain diseases. By presenting this information concisely, it visually reinforces the argument for the necessity of novel, more precise diagnostic tools. The juxtaposition of “Key Clinical Manifestations” with “Key Diagnostic Limitations” directly highlights the diagnostic gaps that epigenetic profiling of CSF cfDNA aims to address, such as non-specificity of existing biomarkers, challenges in genetic testing due to biological complexities (e.g., heteroplasmy), and the late manifestation of definitive symptoms. This structured overview provides a clear foundation for understanding why the proposed liquid biopsy approach may offer a meaningful advance in addressing long-standing diagnostic challenges.

## 4. Cerebrospinal Fluid as a Source for Cell-Free DNA Biomarkers in CNS Pathologies

The utility of liquid biopsy hinges on the accessibility and informative content of circulating biomarkers. Cell-free DNA (cfDNA), comprising fragmented nucleic acids released from cells, primarily through apoptosis, necrosis, or active secretion, is a highly promising analyte [9]. In healthy individuals, cfDNA levels in circulation are typically low (e.g., <10 ng/mL in plasma) and predominantly originate from hematopoietic cells [12]. However, in disease states such as cancer, myocardial infarction, stroke, or diabetes, cfDNA concentrations can significantly increase, sometimes by orders of magnitude, reflecting ongoing cellular damage or turnover [10]. The short half-life of cfDNA, estimated between 4 min and 12 h, allows it to provide a dynamic, “real-time” snapshot of physiological and pathological processes [11].

### 4.1. Unique Characteristics and Functions of Cerebrospinal Fluid

**Figure 7:**
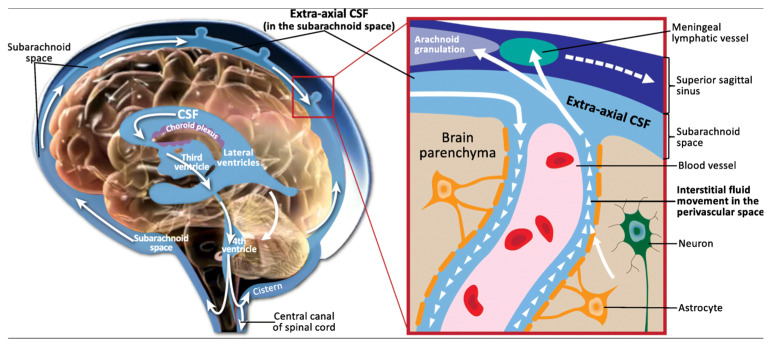
This schematic shows cerebrospinal fluid (CSF) circulation and compartments. CSF is produced in the ventricles, delivering growth factors to neural progenitor cells that form the cortex. It flows through the ventricles into the subarachnoid space (EA-CSF), then circulates into brain tissue via perivascular spaces. Astrocytes aid in clearing waste like amyloid-beta, which exits through lymphatic vessels and arachnoid granulations. The arrow indicates the flow of CSF. This file is licensed under the Creative Commons Attribution 4.0 International license with permission from Wikimedia Commons [49].

Cerebrospinal fluid (CSF) is a clear, colorless liquid that plays a vital role in the protection and physiological maintenance of the central nervous system (CNS) (Figure 7) [24,49]. Primarily produced by the choroid plexus within the ventricular system, with additional contributions from the interstitial space of the brain and the subarachnoid space, CSF circulates throughout the brain and spinal cord [50,51]. Its production is a two-stage process involving passive filtration of plasma through capillary endothelium, driven by osmotic pressure, followed by active transport of plasma ultrafiltrate via choroid plexus epithelial cells, mediated by various ion transporters and aquaporins [51]. This active transport, regulated by enzymes like carbonic anhydrase and specific ion pumps (e.g., Na^+^/K^+^ ATPase), ensures precise control over CSF composition [51].

The functions of CSF are multifaceted. It provides crucial mechanical support, acting as a shock absorber that reduces the effective weight of the brain by 10–15 times, thereby protecting it from sudden impacts [49,51]. Beyond physical protection, CSF is integral to CNS homeostasis, facilitating the transport of essential nutrients, neurotransmitters, and hormones to brain cells while simultaneously eliminating metabolic waste products and toxic substances [49,51]. The integrity of the blood-cerebrospinal fluid barrier (BCB), formed by tight junctions between choroid plexus epithelial cells, is critical for maintaining CSF homeostasis and preventing uncontrolled substance movement [49,51]. Furthermore, CSF serves as an invaluable diagnostic medium, as changes in its composition (e.g., protein levels, cell counts, and the presence of specific biomarkers) are indicative of various CNS diseases, including infections, autoimmune disorders, and neurodegenerative conditions [49,51]. Its direct contact with brain tissue also makes it a conduit for administering drugs that might otherwise be impeded by the blood–brain barrier (BBB) [49,51].

### 4.2. Advantages of CSF-Derived cfDNA over Plasma cfDNA for CNS Pathologies

While plasma cfDNA has proven utility in many systemic diseases, its application for CNS pathologies, particularly brain-specific conditions, is often limited by the blood–brain barrier (BBB) [3]. The BBB is a highly selective physiological barrier that restricts the passage of many molecules, including tumor-derived biomarkers like cfDNA, from the brain into the peripheral bloodstream [3]. Consequently, in primary CNS tumors like gliomas, circulating tumor DNA (ctDNA) levels in plasma are typically very low, often below the detection threshold, making blood-based liquid biopsies challenging for these conditions [3].

**Figure 8:**
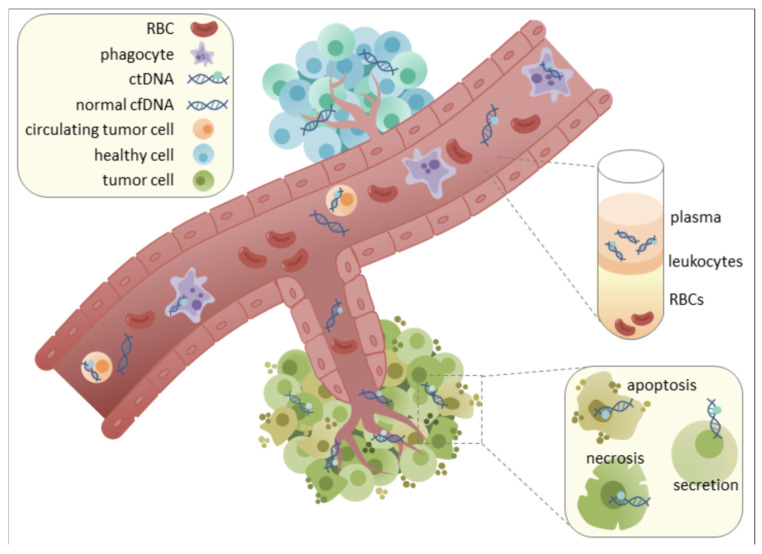
Circulating tumor DNA (ctDNA) is detectable in the serum and plasma components of blood. While the exact mechanism of its release remains unclear, it is thought to result from processes such as apoptosis, necrosis, or active secretion by tumor cells. This file is licensed under the Creative Commons Attribution-Share Alike 4.0 International license with permission from Wikimedia Commons [52].

In stark contrast, CSF offers distinct advantages as a source for CNS-derived cfDNA. Being in direct anatomical contact with the brain and spinal cord, CSF directly reflects the molecular changes occurring within the CNS microenvironment. Studies have shown that the concentration of cfDNA in CSF can be significantly higher than in plasma. For instance, one study reported a mean cfDNA concentration of 26.82 ng/mL in CSF compared to 13.46 ng/mL in plasma [19]. More importantly, the concentration of tumor-specific cfDNA (ctDNA) in CSF can be several orders of magnitude higher than in plasma or urine in CNS malignancies like gliomas (Figure 8) [3,52]. This elevated concentration directly translates to higher sensitivity for the detection of CNS-specific pathologies.

Beyond sheer quantity, the characteristics of cfDNA in CSF also differ from those in plasma. CSF samples exhibit a larger proportion of longer cfDNA fragments (e.g., >200 bp) compared to plasma (37.8% in CSF vs. 22.8% in plasma) [19]. Furthermore, distinct 6 bp end motif patterns allow for the clear separation of cfDNA from CSF and plasma, indicating unique fragmentation processes or cellular origins within each biofluid [19]. CSF is also considered a “less complicated solution system” chemically than plasma, and it contains lower contamination from non-tumor cells, such as peripheral blood cells, which can confound analyses in plasma [3]. This reduced background noise allows for more accurate and reliable detection of CNS-derived signals.

**Figure 9:**
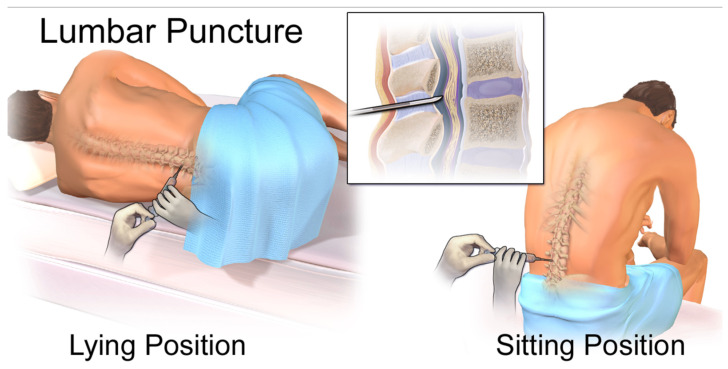
Diagram of a lumbar puncture. This file is licensed under the Creative Commons Attribution 3.0 Unported license with permission from Wikimedia Commons [53].

While lumbar puncture for CSF collection is more invasive than a blood draw, it is a routine clinical procedure that can be performed at the bedside, making longitudinal monitoring feasible for evaluating disease status and treatment response (Figure 9) [3,53]. This direct access to CNS-specific cfDNA, coupled with its higher concentration and lower background contamination, positions CSF as a superior biofluid for liquid biopsy applications in brain diseases, particularly when the blood–brain barrier limits the utility of peripheral blood samples. The distinct fragmentation patterns and end motifs observed in CSF cfDNA also suggest additional layers of information that can be leveraged for the development of advanced biomarkers, providing a richer molecular profile of CNS health and disease.

## 5. Epigenetic Profiling of cfDNA: Fundamental Mechanisms and Methodologies

Epigenetics refers to heritable changes in gene expression that occur without alterations to the underlying DNA sequence [13]. These modifications are crucial for normal development, cell differentiation, and the maintenance of tissue-specific gene expression and cellular identity [13]. Aberrant epigenetic changes are increasingly recognized as fundamental drivers of various human diseases, including cancer and neurodegenerative disorders [13]. Unlike genetic mutations, epigenetic modifications are inherently dynamic and reversible, making them attractive targets for therapeutic intervention and powerful biomarkers for disease detection and monitoring [13].

### 5.1. DNA Methylation: Mechanisms and Role in Gene Regulation

**Figure 10:**
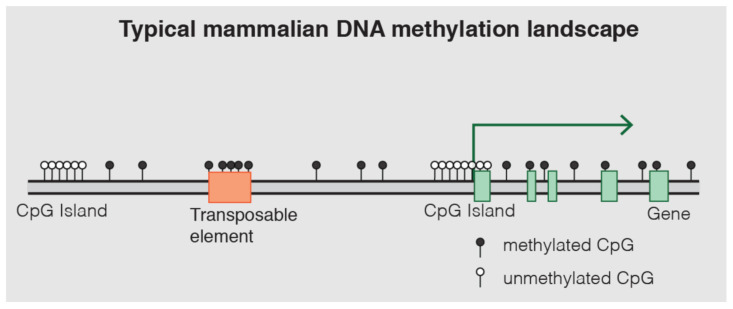
DNA methylation landscape in mammals. This file is licensed under the Creative Commons Attribution-Share Alike 4.0 International license with permission from Wikimedia Commons [54].

DNA methylation is one of the most well-characterized epigenetic mechanisms in mammals [14]. It involves the covalent addition of a methyl group (CH_3_) to the fifth carbon position of a cytosine nucleotide, predominantly occurring within cytosine-guanine (CpG) dinucleotides [13,14,15]. These CpG sites are often clustered in regions known as CpG islands, which are frequently located in gene promoter regions [14]. Approximately 70% of gene promoter regions lie within CpG islands (Figure 10) [14,54].

The process of DNA methylation is dynamically regulated by a balance of “writer” and “eraser” enzymes [55,56]. DNA methyltransferases (DNMTs) are the “writers” responsible for establishing and maintaining methylation patterns. DNMT3A and DNMT3B are de novo methyltransferases that establish new methylation marks on unmethylated CpG sites. At the same time, DNMT1 acts as a “maintenance” methyltransferase, ensuring that methylation patterns are faithfully copied to newly synthesized DNA strands during replication [56]. Conversely, Ten-Eleven Translocation (TET) enzymes act as “erasers”, initiating DNA demethylation by oxidizing 5-methylcytosine (5mC) to 5-hydroxymethylcytosine (5hmC) and further oxidized forms (5fC, 5caC), facilitating both passive (replication-dependent) and active (replication-independent) demethylation pathways [56]. The interplay between DNMTs and TETs sculpts the intricate DNA methylation landscape, which is crucial for the flow of epigenetic information across cell generations [57].

DNA methylation plays a critical role in gene regulation and chromatin structure. When methylation occurs within gene promoter regions, it typically leads to gene silencing. This occurs through two primary mechanisms: methylated cytosines can recruit gene suppressor proteins, and methylation can also induce the formation of heterochromatin. This condensed chromatin state physically prevents the binding of transcription factors and transcriptional machinery to the DNA [11]. In contrast, methylation within gene bodies can be associated with transcript splicing alterations [11]. Aberrant DNA methylation patterns are a hallmark of many diseases. For instance, in cancer, there is often global hypomethylation of the genome, accompanied by focal hypermethylation of tumor suppressor genes, both of which contribute to tumorigenesis [13,14]. Beyond disease, DNA methylation is essential for fundamental biological processes such as tissue-specific gene regulation, genomic imprinting, and X-chromosome inactivation [11]. The unique methylation patterns across different cell types, despite having an identical underlying DNA sequence, form a fundamental aspect of tissue identity and cellular function [13]. This inherent tissue specificity allows DNA methylation patterns to be used to determine the tissue of origin of cfDNA, which is a powerful principle for identifying the source of pathological processes [11].

### 5.2. Methodologies for Methylome Interrogation in cfDNA

Analyzing DNA methylation in cfDNA, particularly from low-input samples such as CSF, requires highly sensitive and specific methodologies. Several advanced techniques have been developed for this purpose.

#### 5.2.1. cfMeDIP-Seq

Cell-free methylated DNA immunoprecipitation and high-throughput sequencing (cfMeDIP-seq) is a method specifically designed for identifying DNA methylation patterns in low-input cfDNA samples [58,59,60,61,62,63,64,65,66]. The technical principle involves isolating cfDNA from the biofluid, followed by shearing the DNA (e.g., via sonication). Antibodies specifically targeting methylated DNA fragments are then used to immunoprecipitate and isolate these methylated regions [66]. The enriched methylated DNA fragments are subsequently identified and quantified using next-generation sequencing (NGS). cfMeDIP-seq offers several advantages [64]. It is not constrained by the need to identify specific tumor mutations; instead, it examines cancer-associated DNA methylation signatures across the entire genome in a tumor-agnostic fashion [64]. Studies have demonstrated its high efficiency and specificity, with cfMeDIP-seq enriching for highly methylated regions, capturing ≥97% of methylated spike-in control fragments with ≤3% non-specific binding [65]. It shows a preference for fragments with higher G + C content and more CpGs, which are often key regulatory regions [65]. This method has been successfully applied to detect various cancers non-invasively, even with minimal sample input, and has shown high sensitivity and specificity in identifying hepatocellular carcinoma (HCC) from plasma cfDNA [64,65]. Its ability to assess peripheral blood plasma from cancer patients without prior knowledge of tumor origin or specific mutations makes it ideal for liquid biopsy applications [64,65].

#### 5.2.2. Targeted Bisulfite Sequencing

**Figure 11:**
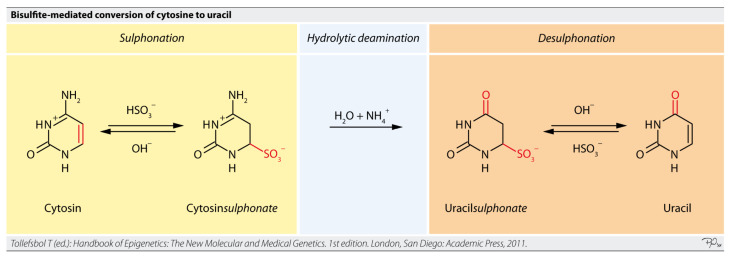
Diagram showing the chemical reaction of the bisulfite-mediated conversion of cytosine to uracil. This file is made available under the Creative Commons CC0 1.0 Universal Public Domain Dedication with permission from Wikimedia Commons [68].

Targeted bisulfite sequencing is an approach that focuses on analyzing DNA methylation in specific regions of interest within the genome, rather than the entire genome (Figure 11) [67,68]. This method combines the fundamental principle of bisulfite conversion with targeted enrichment strategies. Sodium bisulfite treatment converts unmethylated cytosine residues to uracil, while methylated cytosines remain unchanged [61]. During subsequent PCR amplification, uracil is read as thymine, allowing for the differentiation of methylated from unmethylated cytosines by comparing the sequenced reads to a reference genome [67].

**Figure 12:**
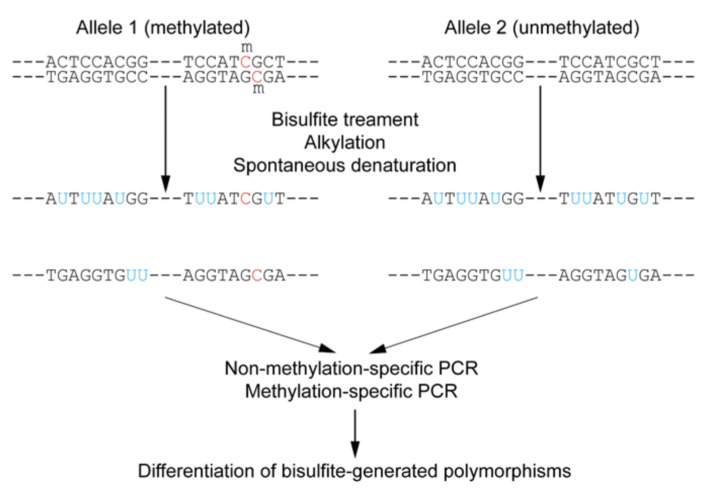
Diagram showing bisulfite sequencing. Permission granted by Wikimedia Commons [69].

Targeted bisulfite sequencing can be performed in various ways. PCR-based enrichment involves designing specific primers to amplify only the target regions of interest (Figure 12) [67,69]. Another variation, Reduced Representation Bisulfite Sequencing (RRBS), enriches for CpG-rich regions by using restriction enzymes (e.g., MspI) to digest genomic DNA, reducing the amount of sequencing required and lowering costs compared to whole-genome bisulfite sequencing (WGBS) [67,70,71,72]. However, RRBS may not cover all CpG regions and is unable to distinguish between 5mC and 5hmC [72]. Oxidative bisulfite sequencing (oxBS-Seq) addresses this by enabling the absolute quantification of both 5mC and 5hmC at single-base resolution [70].

While bisulfite conversion is considered the gold standard for DNA methylation analysis, it has limitations, particularly for ultra-low-input cfDNA. The bisulfite treatment process can cause DNA fragmentation and degradation (up to 30–50% degradation), requiring sufficient DNA input for optimal recovery and potentially leading to lower sequencing quality [4,70,71,72]. Bisulfite-treated DNA is also AT-rich, which can lead to non-specific PCR amplification [70,71,72]. These challenges are particularly pronounced with highly fragmented, low-input cfDNA, where WGBS remains too costly for routine application due to its non-targeted nature and high sequencing budget requirements for robust quantification [4]. RRBS also struggles with highly fragmented or degraded DNA [67,72,73,74].

#### 5.2.3. Enzymatic Methyl-Sequencing (EM-Seq) and EPIC Arrays

Newer enzymatic methods, such as Enzymatic Methyl-sequencing (EM-seq), offer significant advantages over traditional bisulfite conversion. EM-seq uses a milder enzymatic conversion process involving TET2 and APOBEC3A enzymes to convert non-methylated cytosines to uracil, while protecting 5mC and 5hmC from deamination [4]. This enzymatic approach significantly reduces DNA damage and loss (degradation rate < 5%), leading to higher quality and yield of sequencing libraries, especially from limited or degraded DNA samples, such as cfDNA [4]. EM-seq has demonstrated high sensitivity, accurately detecting DNA methylation at single-base resolution with as little as 100 pg of DNA, making it highly suitable for cfDNA analysis in liquid biopsies [74].

DNA methylation arrays, such as the Illumina Infinium MethylationEPIC v2.0 kit, provide a cost-effective and high-throughput alternative for profiling methylation across hundreds of thousands of CpG sites [75]. These microarrays quantitatively interrogate CpGs at single-nucleotide resolution, providing accurate and precise methylation measurements independent of read depth [75]. They cover extensive regions, including CpG islands, genes, and enhancers, and are compatible with various sample types, including formalin-fixed paraffin-embedded (FFPE) tissues, which is valuable for retrospective studies [75]. While arrays offer broad coverage at a lower cost per sample, they provide only partial genomic coverage compared to sequencing methods, potentially missing key methylation sites not included on the array [61].

The choice of methodology for cfDNA methylation analysis depends on the specific research question, available sample input, and desired resolution. While WGBS provides the most comprehensive data, its cost and computational demands are substantial [61]. Targeted approaches like cfMeDIP-seq and targeted bisulfite sequencing offer cost-effectiveness and higher depth in specific regions. Newer enzymatic methods like EM-seq address the critical challenge of DNA degradation in low-input samples, improving data quality and yield. The ongoing advancements in these methodologies are crucial for overcoming the technical hurdles associated with ultra-low-input cfDNA, paving the way for more robust and widespread clinical applications of epigenetic liquid biopsies [76,77].

#### 5.2.4. Emerging Low-Input DNA Methylation Sequencing Methods

Low-input samples (e.g., cell-free DNA from cerebrospinal fluid) demand highly sensitive and DNA-preserving methylation assays. Table 2 summarizes various sequencing method across key parameters: sensitivity, cost, resolution, DNA input requirements, and compatibility with CSF cfDNA. Several recently developed methods address these needs:

- TAPS (TET-assisted pyridine borane sequencing): This is a bisulfite-free technique that uses enzymatic oxidation and mild chemical reduction to detect 5-methylcytosine (5mC) directly. TAPS avoids the harsh bisulfite treatment, thus preserving DNA integrity and sequence complexity. Only methylated cytosines are converted to thymine, minimizing DNA fragmentation and mapping bias. This yields higher sequencing quality, improved read mapping, and lower cost compared to traditional bisulfite sequencing.
- UBS-seq (Ultra-fast Bisulfite Sequencing): This is an optimized bisulfite sequencing protocol tailored for ultra-low DNA inputs. UBS-seq uses highly concentrated bisulfite and high temperature for a very short duration (~10 min) to achieve complete C → U conversion. The much shorter treatment time dramatically reduces DNA degradation and false conversions, producing ~20-fold lower background noise than conventional bisulfite sequencing. With less bias and damage, UBS-seq enables accurate base-resolution methylation mapping from extremely small DNA amounts (even single cells or just a few nanograms of cfDNA).
- LABS (Linear Amplification-Based Bisulfite Sequencing): LABS is a recently introduced method that replaces PCR with linear pre-amplification to build sequencing libraries from bisulfite-converted DNA. By amplifying DNA fragments in a linear, unbiased manner, LABS avoids the selective loss of rare molecules often seen in exponential (PCR) amplification. LABS has demonstrated improved detection of tumor-specific methylation signals in cfDNA, enhancing sensitivity for liquid biopsies where DNA is scarce.
- Nanopore-based methylation detection: Long-read nanopore sequencing (e.g., Oxford Nanopore Technologies) can directly identify methylated bases on single DNA molecules in real time. As DNA passes through the nanopore, methylation alters the electrical current trace, enabling base-resolution 5mC detection without bisulfite conversion or PCR. This single-molecule approach preserves native DNA (no chemical damage) and simultaneously reads genetic and epigenetic information. Nanopore sequencing has been used to profile methylation in minute cfDNA quantities (on the order of nanograms), successfully detecting differentially methylated regions in low-abundance cell-free samples. Although error rates are higher than short-read methods, ongoing improvements (e.g., higher consensus accuracy and machine learning signal processing) are enhancing its sensitivity and reliability for low-input methylation analysis. Although error rates are higher than short-read methods, ongoing improvements (e.g., higher consensus accuracy and machine learning signal processing) are enhancing its sensitivity and reliability for low-input methylation analysis.

### 5.3. Other Epigenetic Mechanisms: Histone Modifications and Non-Coding RNAs

Beyond DNA methylation, additional epigenetic layers contribute to the regulation of gene expression and should be considered for a comprehensive overview. Histone modifications are a key epigenetic mechanism involving chemical alterations of histone proteins (such as acetylation, methylation, phosphorylation, and ubiquitination) that affect how tightly DNA is packaged into chromatin. Importantly, histone modifications can also be probed in the context of cfDNA. Because apoptotic cfDNA is released wrapped around nucleosomes, the positions and protection patterns of these fragments carry information about nucleosome positioning and associated histone marks. Recent studies have even demonstrated that tissue-specific histone marks leave characteristic footprints in cfDNA fragmentation patterns: for example, nucleosomes bearing H3K27ac (a mark of active enhancers) in liver or placenta cells produce distinct cfDNA fragment signatures that can reveal contributions of those tissues to the circulating DNA Such approaches, which combine cfDNA “fragmentomics” with knowledge of histone modification landscapes, have been used to improve cancer detection and to deduce the tissue of origin of cfDNA in plasma. This integration of histone modification profiling into liquid biopsy analyses holds promise for complementing DNA methylation-based biomarkers with additional information about gene regulatory activity in health and disease.

Another crucial epigenetic layer involves non-coding RNAs (ncRNAs), which encompass a diverse group of RNA molecules (including microRNAs, small interfering RNAs, and long non-coding RNAs) that do not encode proteins but can modulate gene expression. NcRNAs are now recognized as significant regulators of the epigenome. The dysregulation of ncRNAs is linked to many diseases (notably cancer, where tumor-suppressive miRNAs are often downregulated and oncogenic miRNAs are upregulated), underscoring their role in epigenetic networks. In the context of liquid biopsies, circulating ncRNAs (particularly microRNAs) have been widely explored as potential biomarkers that reflect underlying pathophysiological processes. These cell-free RNAs, often encapsulated in exosomes or other vesicles, can be detected in biofluids and may provide complementary information to cfDNA. For example, multi-analyte diagnostic panels that combine circulating miRNA profiles with cfDNA and protein markers have shown improved accuracy for cancer detection compared to single-marker approaches. Such findings highlight that ncRNA-based insights can augment the epigenetic profiling of diseases.

### 5.4. Current Status of DNA Methylation Sequencing Methods

cfMeDIP-seq (cell-free methylated DNA immunoprecipitation sequencing) has moved into translational use. It enables genome-wide cfDNA methylation profiling from ~1–10 ng inputs and has been applied in numerous patient studies (e.g., early cancer detection and tumor subtyping). For instance, cfMeDIP-seq has been used to detect distinctive methylation patterns in liquid biopsies for intracranial brain tumors, to differentiate localized vs. metastatic prostate cancer, and to monitor minimal residual disease. Targeted bisulfite sequencing of known methylation markers is also well-established in clinical research. A prominent example is the GRAIL Galleri multi-cancer blood test, which uses targeted bisulfite-based methylation panels and has been evaluated in large clinical trials. The SYMPLIFY study reported ~66% overall sensitivity at >98% specificity. Illumina EPIC methylation arrays (850K CpG microarrays) have been piloted on low-input cfDNA for biomarker discovery—for example, one proof-of-concept EPIC study profiled plasma cfDNA from metastatic breast cancer patients and identified a 1467-CpG “episignature” (including WNT1 hypermethylation) that distinguished cases from controls. Newer bisulfite-free methods are now emerging to improve sensitivity with minimal DNA. TAPS (TET-assisted pyridine borane sequencing) is a 5mC/5hmC sequencing technique that avoids DNA damage; notably, deep whole-genome cfDNA TAPS was recently used in a multi-cancer diagnostic study, achieving ~94.9% sensitivity at 88.8% specificity and the authors deem the platform “ready for further clinical evaluation”. Likewise, EM-seq (enzymatic methyl sequencing) replaces harsh bisulfite conversion with enzymatic steps, preserving cfDNA integrity and allowing lower inputs. This approach has been integrated into translational assays (e.g., HelioHealth’s colorectal screening panel uses targeted EM-seq) and can even capture additional fragmentomic signals (like nucleosome positioning) from the same library. Direct nanopore sequencing of methylation is also being explored for ultra–low-input samples: a 2024 study demonstrated that Oxford Nanopore sequencing of cfDNA from CSF could classify pediatric brain tumors, underscoring its promise for clinical brain tumor diagnostics and disease monitoring.

In contrast, some methods remain at a preclinical or developmental stage for cfDNA/CSF applications. Standard RRBS (reduced-representation bisulfite sequencing) is ill-suited to already-fragmented cfDNA—its restriction/size-selection strategy, designed for intact genomic DNA, fails to enrich CpG islands when applied to cfDNA. An adapted protocol (cfRRBS) has been proposed to overcome this; in a pilot, cfRRBS achieved ~94% accuracy classifying pediatric tumors from plasma cfDNA and is being tested on CSF samples, but this remains a research tool. OxBS-seq (oxidative bisulfite sequencing for 5hmC) also sees limited translational use due to its high DNA input needs and damage: like other bisulfite-based assays it causes severe DNA degradation and complexity loss, so it has been used mainly for exploratory 5hmC mapping in lab settings. UBS-seq (“ultrafast” bisulfite sequencing) accelerates and intensifies bisulfite chemistry to reduce DNA damage, enabling library prep from tiny samples (e.g., single-digit nanograms of cfDNA or even 1–100 cells) with higher CpG coverage than conventional BS-seq. Similarly, LABS (linear amplification–based bisulfite sequencing) forgoes standard PCR in favor of linear amplification, boosting sensitivity to trace methylation signals. In 2024 its developers demonstrated genome-wide methylation and copy-number profiling from <1 ng cfDNA: applying LABS to ~100 patient plasma samples revealed cancer-specific methylation patterns and improved tissue-of-origin resolution. These cutting-edge methods (UBS-seq, LABS) have shown technical feasibility on clinical specimens, but they are not yet in routine trials or practice—their use so far is confined to methodological studies and proof-of-concept validations in the lab.

## 6. Translational Applications: Mapping Brain Cell-Type-Specific Injury, Inflammatory Signaling, and Metabolic Reprogramming

The ability to profile epigenetic signatures in CSF-derived cfDNA opens unprecedented avenues for understanding the molecular underpinnings of metabolic brain diseases. This approach moves beyond traditional bulk tissue analysis, offering the potential to resolve disease processes at the level of specific brain cell types, track inflammatory responses, and identify metabolic reprogramming events.

### 6.1. Brain Cell-Type-Specific Injury

A profound strength of cfDNA methylation profiling lies in its capacity to identify the tissue and even cell type of origin of circulating DNA fragments [11]. Each cell type in the body possesses a unique DNA methylation pattern, which is intrinsically linked to its gene expression profile and cellular identity [11]. These methylation patterns are remarkably stable under physiological and pathological conditions, allowing them to serve as reliable markers of cell death in specific organs [15]. When cells in a particular brain region or of a specific cell type undergo damage or death, they release their DNA into the CSF, carrying these unique methylation signatures [9].

**Figure 13:**
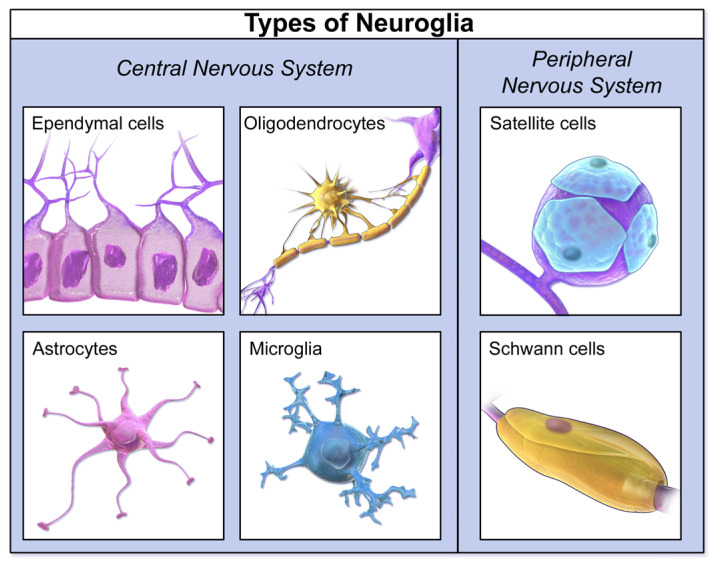
Types of Neuroglia. This work is free and may be used by anyone for any purpose with permission from Wikimedia Commons [85].

Sophisticated deconvolution algorithms have been developed to analyze the mixed cfDNA methylation profiles in CSF and estimate the proportional contributions from different brain cell types, such as neurons, astrocytes, oligodendrocytes, and microglia [11]. For instance, studies have shown that cfDNA methylation changes can reflect cell-type-specific gene dysregulation in the brains of patients with neurodegenerative diseases, such as Amyotrophic Lateral Sclerosis (ALS), particularly in excitatory neurons and astrocytes [78,79,80]. In Alzheimer’s disease (AD), specific DNA methylation signatures initially identified in cortical neurons and brain tissue have been validated in CSF-derived cfDNA, demonstrating the potential to diagnose AD even before clinical manifestation [78,81,82,83,84]. This capability to detect cfDNA originating from minority cell populations, such as oligodendroglial lineage cells in multiple sclerosis, highlights the exquisite sensitivity of this approach (Figure 13) [11,81,82,83,84,85].

The ability to pinpoint the specific brain cell types undergoing injury or death offers a granular understanding of disease progression that is unattainable with bulk tissue analysis or traditional neuroimaging. This is particularly relevant for metabolic brain diseases, where different cell populations (e.g., neurons, glia) may be differentially affected by metabolic defects. For example, in GLUT1DS, understanding which specific brain cells are most impacted by glucose deficiency could refine therapeutic strategies. Similarly, in neurodegenerative conditions like frontotemporal dementia (FTD), cfDNA methylation changes in CSF have been observed to be dependent on the clinical stage and can even show subtype-specific patterns, providing clues about the genomic location of these changes and their tissue origin [82]. This level of detail allows for a more precise mapping of localized neurodegeneration and provides valuable insights into the underlying pathological mechanisms.

Neurons and glial cells (astrocytes, oligodendrocytes, and microglia) exhibit inherently different DNA methylation landscapes under normal conditions, reflecting their specialized roles. Neuronal DNA is marked by distinctive epigenetic features, notably abundant non-CpG (CpH) methylation and elevated levels of 5-hydroxymethylcytosine (5hmC), which are largely absent in adult glial cells. Neurons also tend to maintain higher global CpG methylation levels than glia, whereas glial genomes show focal hypermethylation at certain loci (often silencing neuron-specific genes that remain hypomethylated in neurons). Importantly, many neuron–glia methylation differences localize to cell-type-specific enhancers and regulatory regions, underscoring how DNA methylation helps lock in each cell type’s gene expression program.

Disease states can disrupt these methylation landscapes in cell-type-specific ways, mirroring each cell type’s susceptibility and role in pathology. For example, in Alzheimer’s disease, chronic neuroinflammation drives epigenetic remodeling in glia: activated microglia and reactive astrocytes show aberrant DNA methylation changes at pro-inflammatory gene promoters (e.g., TNF-α, IL-1β, IL-6), unleashing sustained cytokine expression that exacerbates pathology. Neurons in AD, meanwhile, may accumulate methylation changes at genes related to synaptic plasticity and survival, reflecting their vulnerability to amyloid and tau toxicity. In ALS, the relatively selective death of motor neurons is accompanied by both intrinsic and extrinsic epigenetic alterations—degenerating neurons show DNA methylation abnormalities (e.g., 5mC accumulation in motor neuron genomes). However, surrounding astrocytes and microglia undergo reactive epigenomic changes linked to neurotoxic inflammation. Notably, methylation profiling of ALS patient samples reveals that the disease perturbs gene regulation in specific cell types, particularly excitatory neurons and astrocytes. Even metabolic and genetic disorders such as MELAS (a mitochondrial encephalopathy) or lysosomal storage diseases elicit divergent epigenetic responses in neurons versus glia. Energy-deficient neurons in MELAS may exhibit aberrant methylation due to impaired metabolic enzymes and heightened oxidative stress, whereas in lysosomal storage disorders microglia become engorged with undigested substrates and respond by activating inflammatory and clearance pathways—a process accompanied by profound transcriptional and epigenetic changes in these glial cells. Thus, each cell type’s DNA methylation landscape is reshaped in disease according to its unique stressors—with neurons often showing methylation changes in pathways of excitability, metabolism, or survival, and glial cells showing changes in immune or homeostatic pathways—reflecting their differential susceptibility and contributions to disease progression.

### 6.2. Inflammatory Signaling

Neuroinflammation is a common pathological feature across various neurological disorders, including metabolic brain diseases, and contributes significantly to neuronal damage and cognitive decline [45]. Epigenetic mechanisms, particularly DNA methylation, play a crucial role in regulating inflammatory gene expression and signaling pathways within the CNS [83].

In Niemann-Pick disease type C1 (NPC1), for instance, neuroinflammation is a prominent part of the pathological cascade [86]. Studies in NPC1 mouse models and human post-mortem brain tissues have identified significant changes in the expression of inflammation-associated genes, such as complement 3 (C3) [86]. Analysis of CSF from NPC1 patients has revealed altered levels of various inflammatory markers, including increased interleukin 3 (IL-3), chemokine (C-X-C motif) ligand 5 (CXCL5), interleukin 16 (IL-16), and chemokine ligand 3 (CCL3), alongside decreased levels of anti-inflammatory cytokines like IL-4, IL-10, and IL-13 [86]. These findings suggest that CSF-derived cfDNA methylation patterns could reflect these inflammatory shifts, providing a molecular readout of neuroinflammation.

Similarly, in neuronopathic Gaucher disease (nGD), neuroinflammation is a central pathological feature driven by the accumulation of glycosphingolipids and the activation of microglia, NK cells, astrocytes, and neurons [87]. Targeted genetic rescue experiments have demonstrated that ameliorating the buildup of glucosylceramide (GlcCer) and glucosylsphingosine (GlcSph), particularly in microglia and macrophages, can reverse neuroinflammation and improve survival [87,88,89,90]. This highlights the critical role of specific cell types in driving the inflammatory response. In diabetes-associated cognitive decline (DCD), hyperglycemia itself triggers a neuroinflammatory state by promoting the formation of advanced glycation end products (AGEs) and inducing oxidative stress, leading to the upregulation of inflammatory cytokines (e.g., TNF-α, IL-1, IL-2, IL-6) and damage to the blood–brain barrier [45]. Sustained glial activation and inflammation can make the brain more susceptible to injury and neurodegeneration [45].

Epigenetic profiling of CSF cfDNA offers a unique opportunity to monitor these inflammatory signaling pathways. Changes in DNA methylation patterns on cfDNA fragments originating from activated microglia or astrocytes, or neurons undergoing inflammatory stress, could serve as sensitive biomarkers of neuroinflammation. Given that cfDNA levels themselves can be influenced by inflammation and can trigger immune-inflammatory reactions, analyzing the epigenetic landscape of CSF cfDNA could provide a comprehensive picture of the inflammatory status within the CNS, aiding in disease monitoring and evaluating the efficacy of anti-inflammatory therapies (Table 2) [12].

### 6.3. Metabolic Reprogramming

Metabolic reprogramming, the alteration of cellular metabolic pathways, is a fundamental process in many diseases, including metabolic brain disorders. Epigenetic modifications are intricately intertwined with cellular metabolism, as metabolites can serve as substrates or cofactors for epigenetic modifying enzymes, and conversely, epigenetics can regulate the expression of metabolic genes [55].

In GLUT1 Deficiency Syndrome, the primary metabolic defect is an insufficient transport of glucose into the brain [40]. Beyond the direct energy deficit, this chronic neuroglycopenia can cause broader disruptions at the cellular level. Next-generation metabolic screening of CSF from GLUT1DS patients has identified novel biomarkers, including decreased levels of gluconic and galactonic acid, as well as xylose-α1-3-glucose and xylose-α1-3-xylose-α1-3-glucose [34,35,36]. The latter two, potentially originating from O-glycosylated proteins, suggest that insufficient glucose may affect protein glycosylation, impacting various cellular processes beyond just energy production [34,35,36]. These findings imply that brain glucose deficiency leads to metabolic reprogramming, which could be reflected in cfDNA methylation patterns (Table 2).

In diabetes-associated cognitive decline (DCD), impaired glucose homeostasis and insulin signaling play a central role in brain dysfunction [44]. Chronic hyperglycemia and insulin resistance induce oxidative stress, neuroinflammation, and impaired synaptic plasticity, all of which contribute to cognitive impairment [41]. The brain’s reliance on glucose catabolism for energy production and neurotransmission means that metabolic modifications, particularly those affecting glucose oxidation pathways and the glutamine-glutamate/GABA cycling between astrocytes and neurons, are crucial for functional deterioration [44]. Epigenetic modifications are known to be influenced by various extrinsic factors, including nutrition and metabolic state [55]. For instance, maternal obesity has been linked to altered offspring brain DNA methylation profiles and behavior, detectable through cell-free fetal DNA (cffDNA) methylation in maternal blood, correlating with metabolic and immune markers [88,89,90]. This demonstrates a clear link between systemic metabolic state, epigenetic changes, and neurodevelopment (Table 2).

CSF cfDNA methylation profiling can provide insights into these metabolic reprogramming events within the brain. By identifying specific methylation patterns associated with altered glucose utilization, mitochondrial dysfunction, or shifts in metabolic pathways (e.g., one-carbon metabolism, glycolysis/OXPHOS switch) in specific brain cell types, this approach can uncover the molecular signatures of metabolic stress and adaptation [27,28,88,89,90,91,92,93,94,95,96,97,98,99]. This offers a powerful means to understand how metabolic defects translate into neurological dysfunction and to monitor the efficacy of interventions aimed at restoring metabolic balance in the brain (Table 2).

## 7. Diagnostic and Prognostic Utility of CSF cfDNA Methylation Biomarkers

The inherent properties of cfDNA, combined with the specificity of epigenetic signatures, position CSF cfDNA methylation profiling as a powerful tool with significant diagnostic and prognostic potential for metabolic brain diseases. This approach offers advantages over traditional CSF markers and neuroimaging, particularly in early detection and dynamic monitoring (Table 2 and Table 4).

There exist promising clinical applications of CSF cfDNA methylation regarding previously discussed metabolic brain disorders, although as of now these patterns are correlatory of existence of the disease and there is not a clear correlation between degree of epigenetic modulation and degree of clinical manifestation. In Niemann-Pick Disease, for example, NPC1 mice exhibit reduced DNA methyltransferase levels and global hypomethylation due to cholesterol disruption of folate/methylation pathway. In Gaucher disease, Gaucher patients had histone H4 hypoacetylation correlating with reduced neurotrophic factor expression due to glucocerebrosidase lack of function. In diabetes-related cognitive decline, mouse models exhibit global DNA hypermethylation in neurons that silences neuroprotective genes—the promoter methylation–mediated suppression of HSF1 (heat shock factor 1). However, treating diabetic neurons with a DNA methylation inhibitor reactivated HSF1 and heat-shock proteins, while reducing astroglial inflammation (GFAP) and cytokine level. These findings also indicate that diabetes-induced methylation changes actively contribute to cognitive decline by turning off genes for synaptic plasticity and stress response, rather than being mere byproducts.

### 7.1. Comparison with Traditional CSF Markers and Neuroimaging

Traditional CSF markers for neurological diseases typically include protein levels, cell counts, glucose, and lactate, which provide general indicators of infection, inflammation, or metabolic disturbances [81,89,90,91,92,93,94,95,96]. For specific MBDs, markers like elevated CSF lactate in MELAS are indicative but lack specificity [21,24,25]. In GLUT1DS, low CSF glucose and lactate are characteristic, but these levels can be borderline or near normal in mild cases, limiting their diagnostic accuracy [34,35,36]. Neuroimaging techniques, such as MRI and PET, provide structural and functional insights into the brain. While MRI can reveal stroke-like lesions in MELAS or atrophy in advanced LSDs, these findings are often nonspecific or absent in early disease stages [34,35,36]. PET scans can show reduced brain metabolism in GLUT1DS or DCD, but their reliability for definitive diagnosis is not fully established (Table 1 and Table 2) [3,4,5,6,7,8,9,10,11,12,13,14,15,16,17,18,19,20,21,22,23,24,25,26,29,30,31,32,33,34,35,36,39,40].

CSF cfDNA methylation biomarkers offer several distinct advantages. Firstly, they provide molecular-level information directly from the CNS, reflecting cell death and gene regulatory changes that are often more specific to disease pathology than general metabolic markers or structural imaging [9]. The ability to identify the tissue and cell type of origin of cfDNA fragments allows for precise mapping of CNS damage, which is a significant leap beyond bulk CSF protein measurements or broad imaging findings [11]. For instance, while neurofilament light chain (NfL) is a valuable general biomarker of neurodegeneration, it lacks the targeted insights that cfDNA methylation can provide regarding specific cell populations [78] (Table 1 and Table 2).

Secondly, cfDNA has a short half-life (minutes to hours), making it a dynamic biomarker that can provide a “real-time” assessment of disease activity and treatment response [11]. This contrasts with some traditional protein biomarkers or neuroimaging findings that may represent late-stage pathology or accumulate slowly [15]. For example, in Alzheimer’s disease, while amyloid-beta changes are noted early, pTau levels, crucial for differential diagnosis, often increase only in advanced stages [81]. Epigenetic cfDNA analysis can potentially detect early, subtle molecular shifts before overt clinical symptoms or significant structural changes are visible, thereby expanding the therapeutic window (Table 1 and Table 2) [9].

### 7.2. Diagnostic and Prognostic Applications in Neurometabolic Disorders

The application of CSF cfDNA methylation profiling holds substantial promise for improving the diagnosis and prognosis of metabolic brain diseases (Table 3).

- Early Detection and Differential Diagnosis: The capacity of cfDNA methylation to identify specific tissue and cell-type signatures means it can potentially detect early cellular damage or dysfunction in the brain before widespread clinical symptoms emerge [16]. This is particularly critical for MBDs, where early intervention can significantly impact disease progression and patient outcomes [2]. Moreover, by providing highly specific molecular fingerprints, cfDNA methylation patterns can aid in differentiating between various MBDs that share common, non-specific clinical presentations, or distinguish them from other neurological disorders they mimic [11]. For example, in CNS tumors, methylation-based classification of CSF cfDNA has shown high accuracy in discriminating major malignant brain tumor types, approaching the accuracy of standard-of-care tissue biopsies [93]. While MBDs are not tumors, this demonstrates the principle of specific classification.
- Monitoring Disease Progression and Treatment Response: The dynamic nature of cfDNA, with its short half-life, allows for repeated sampling and real-time monitoring of disease activity [9]. Changes in the levels or methylation patterns of cfDNA originating from specific brain cell types could indicate progression of neurodegeneration, shifts in inflammatory states, or the effectiveness of therapeutic interventions [9]. This is valuable for assessing the impact of therapies aimed at correcting metabolic defects or mitigating neuroinflammation. For instance, in glioma, longitudinal CSF cfDNA monitoring has shown changes in tumor-associated variant allele frequencies in response to chemoradiation, even through pseudoprogression [95]. This principle can be extended to MBDs to track the molecular response to treatments.
- Prognostic Insights: Elevated cfDNA concentrations, particularly of circulating tumor DNA (ctDNA) in oncology, often correlate with overall tumor burden and advanced disease stage, indicating poorer clinical outcomes [4]. While direct correlations for MBDs are still emerging, it is plausible that specific cfDNA methylation signatures or quantitative changes could provide prognostic information, predicting disease severity, progression rates, or response to specific therapies. This could enable more precise risk stratification and personalized treatment planning for patients with MBDs (Table 2) [9].

The integration of CSF cfDNA methylation analysis into clinical evaluation holds the potential to transform the diagnostic paradigm for metabolic brain disorders. By offering a minimally invasive window into the molecular pathology of the CNS, it can facilitate earlier, more accurate diagnoses, enable dynamic disease monitoring, and provide critical prognostic insights, ultimately paving the way for more targeted and effective patient management strategies (Table 4).

### 7.3. Comparison of CSF cfDNA Methylation Profiling Vs. Conventional Diagnostic Workups

Regarding the timing of diagnostic workups, for example of MBDs, diagnostics often require multiple sequential tests that can prolong the “diagnostic odyssey”. Neuroimaging and basic biochemical assays (e.g., serum/CSF metabolites) can yield results rapidly (hours to days), whereas confirmatory enzyme or genetic tests may take weeks. CSF cfDNA methylation analysis is not yet routine, but in concept it could provide real-time insight into ongoing CNS injury. Early studies suggest that epigenetic signals in CSF may appear before overt changes are detectable by imaging or traditional markers, potentially flagging pathology at an earlier stage.

Each modality offers different degrees of specificity. Standard CSF metabolite analyses (e.g., lactate, amino acids) are sensitive clues but often not disease-specific—for example, elevated lactate in CSF is common in mitochondrial encephalopathies but not specific to MELAS and occurs in other conditions or even may be normal in some confirmed cases. Likewise, MRI findings (such as white-matter changes or “stroke-like” lesions) and MRS metabolite peaks can be suggestive (e.g., a brain lactate peak on MRS in mitochondrial disease, or a characteristic hypometabolism pattern on PET in GLUT1 deficiency. Enzyme assays target a known deficiency (highly specific for that enzyme’s disorder), but false positives can occur with related conditions (e.g., elevated oxysterol levels in Niemann–Pick C must be interpreted cautiously, as they also rise in other lipid storage diseases. CSF cfDNA methylation profiling, by contrast, leverages the inherent tissue- and cell-type specificity of DNA methylation patterns. This means cfDNA signatures could pinpoint a CNS origin and even distinguish neuronal vs. glial DNA release, adding specificity about the tissue affected that metabolite levels or systemic DNA tests cannot provide. In summary, traditional tests often identify a broad metabolic disturbance, while cfDNA methylation could enhance specificity by revealing the precise CNS involvement or even disease-associated epigenetic “fingerprints”. This specificity may help differentiate MBDs, although it remains under investigation.

From a practical standpoint, conventional approaches are well-established and widely available, whereas CSF cfDNA methylation profiling is still experimental. Clinicians investigating a suspected MBD will nearly always proceed with standard tests—metabolic panels, MRI/MRS, enzyme assays, targeted gene panels or whole exome sequencing—because these have defined clinical utility and guideline recommendations. By contrast, cfDNA methylation analysis currently requires advanced laboratory techniques (e.g., bisulfite sequencing or cfMeDIP-seq) and bioinformatics expertise not found in routine practice. The cost and infrastructure needed are significant, and results are not instantly available. Moreover, there are no formal guidelines yet that include cfDNA-based diagnostics for metabolic brain disorders; the approach remains in the research realm as of 2025.

If cfDNA profiling proves its value, it could be integrated into the diagnostic workflow by using the same CSF sample already taken for metabolites, minimizing additional burden. Its greatest near-term utility might be in complex cases where standard tests are inconclusive—for instance, a child with signs of a neurometabolic disorder but no definitive genetic diagnosis could have CSF sent for cfDNA methylation analysis under research protocols to look for otherwise hidden clues. In summary, traditional methods currently drive diagnosis and will continue to do so due to accessibility and proven merit, while cfDNA methylation assays represent an emerging adjunct that needs further validation before routine use.

## 8. Challenges and Future Directions in Epigenetic Liquid Biopsy for Neurometabolic Disorders

Despite the immense promise of epigenetic profiling of CSF-derived cfDNA for metabolic brain diseases, several technical, interpretive, and clinical challenges must be addressed to facilitate its widespread clinical translation.

### 8.1. Technical Challenges

The analysis of ultra-low-input cfDNA, particularly from CSF, presents significant technical hurdles.

- Low DNA Concentration: While CSF cfDNA concentrations can be higher than plasma for CNS pathologies, the absolute quantities remain low compared to traditional tissue biopsies [5]. This low input can compromise the sensitivity and reliability of methylation assays, increasing the risk of false-positive results [4,67]. Methods like cfMeDIP-seq and enzymatic methyl-sequencing (EM-seq) are designed for low-input samples, but consistent high-quality data remains a challenge [64]. Ultra-low DNA inputs often require additional PCR amplification to generate sequencing libraries, but this step can introduce bias and uneven genomic coverage. When starting material is scarce, certain fragments may be preferentially amplified while others drop out, yielding an incomplete representation of the methylome. These factors together mean that key CpG sites or loci might be missed due to stochastic sampling errors. Approaches such as incorporating unique molecular identifiers and optimizing library preparation protocols (e.g., single-stranded library prep or milder enzymatic conversion) are being explored to mitigate PCR-induced artifacts, but achieving uniform, comprehensive methylation coverage from minimal CSF cfDNA remains challenging
- DNA Integrity and Degradation: Bisulfite conversion, a cornerstone of many methylation analysis methods, can cause significant DNA fragmentation and degradation, impacting sequencing quality and accuracy [4]. While EM-seq offers a milder, less degradative alternative, optimizing library preparation for highly fragmented cfDNA is crucial [67].
- Contamination and Background Noise: Although CSF generally has lower non-tumor cfDNA contamination than plasma, contamination from cellular DNA during sample collection or processing can still occur [3,5]. This can introduce high molecular weight DNA contamination, affecting the accuracy of methylation classification [5]. Careful sample handling protocols, including immediate centrifugation and proper storage, are essential to minimize this [11].
- Limited Cell-of-Origin Resolution: Disease-relevant methylation signals from rare CNS cell populations (e.g., specific neuron or glia subtypes) can be diluted by more abundant DNA from other sources, making it hard to detect subtle, cell-specific patterns. Current deconvolution algorithms can infer cell-type contributions, but their accuracy is constrained by the need for robust reference methylation maps for diverse brain cell types (many of which are still being refined). In practice, this limited resolution means that some epigenetic biomarkers might be obscured by background noise or misattributed, underscoring the technical need for more sensitive methods and comprehensive reference datasets to pinpoint the origins of CSF cfDNA methylation signals.
- Standardization of Assays: A major barrier to clinical adoption is the lack of standardized protocols for cfDNA collection, processing, and analysis [11]. Variability in laboratory procedures, from DNA extraction kits (e.g., MagMAX) to library preparation (e.g., SureSelect XT HS2) and sequencing platforms (e.g., Illumina NovaSeq), can lead to inconsistent results across studies and laboratories [5]. Specifically, differences in CSF sample collection and handling significantly influence cfDNA yield and quality. Delays in processing, suboptimal storage temperatures, or repeated freeze–thaw cycles may degrade cfDNA or introduce cellular DNA contamination, confounding downstream methylation analysis. Additionally, immediate processing (prompt centrifugation, cold storage) and standardized handling are essential, as inconsistent pre-analytical protocols can lead to variability in cfDNA concentrations and fragment profiles. Establishing robust, reproducible, and universally accepted protocols is critical for validating cfDNA methylation biomarkers for routine clinical use [77].

### 8.2. Interpretive Challenges

The complexity of cfDNA methylation data necessitates advanced bioinformatics and careful interpretation.

- Data Complexity and Bioinformatics: Genome-wide methylation profiling generates vast, high-dimensional datasets that require significant computational resources and specialized bioinformatics expertise for processing, analysis, and interpretation [4]. Developing user-friendly software tools and pipelines that can accurately deconvolve tissue-of-origin signals from complex cfDNA mixtures and identify disease-specific methylation patterns is an ongoing challenge [92].
- Cell-Type Deconvolution Accuracy: While deconvolution algorithms (e.g., CelFiE, CelFEER, cfDecon) are advancing, accurately estimating cell-type proportions from cfDNA methylation data, especially for rare cell types or in the presence of unknown cell types, remains challenging [92]. The accuracy of these methods relies on robust reference methylome datasets for various brain cell types, which are still being developed [17].
- Dynamic Nature of Epigenetic Signatures: Epigenetic signatures can change throughout disease progression or in response to treatment [11]. While this dynamism offers opportunities for monitoring, it also adds complexity to interpretation, as a single methylation profile may not capture the full disease trajectory. This necessitates longitudinal studies and the development of dynamic predictive models.
- Overfitting and Generalizability: Methylation classifiers, especially for rare diseases or subtypes, can suffer from overfitting if training datasets are not comprehensive or representative [61]. This can limit their generalizability to new, unseen patient populations, potentially leading to misclassification [61].

### 8.3. Clinical Integration and Regulatory Challenges

Translating epigenetic liquid biopsy from research to routine clinical practice involves significant regulatory, ethical, and economic considerations.

- Invasiveness of CSF Collection: While less invasive than brain biopsy, lumbar puncture for CSF collection is still an invasive procedure that can cause discomfort and carries risks such as post-dural puncture headache [5]. This limits its applicability for widespread screening or very frequent monitoring, particularly in pediatric populations where ethical standards are stricter [6].
- Regulatory Frameworks: The regulatory landscape for novel liquid biopsy biomarkers, especially for non-oncological CNS conditions, is still evolving [6]. Clear guidelines for assay validation, clinical utility, and reimbursement are necessary to accelerate adoption. The FDA’s emphasis on clear scientific rationale, risk minimization, and comprehensive informed consent for biopsies in clinical trials underscores the stringent requirements for new diagnostic tools [6].
- Cost-Effectiveness: The advanced technologies required for cfDNA methylation analysis, such as next-generation sequencing, can be expensive, with costs primarily driven by consumables [4]. While liquid biopsies can offer downstream economic benefits by avoiding more invasive procedures or guiding more efficient therapy allocation, the initial cost remains a significant barrier to widespread clinical use, particularly for rare diseases [11]. Developing more cost-effective molecular profiling methods and non-proprietary assay panels is crucial [11].
- Ethical Considerations: Beyond the invasiveness of CSF collection, data privacy concerns and ethical issues related to genetic and epigenetic information derived from liquid biopsies need careful consideration, especially as these technologies become more accessible [99].

### 8.4. Future Directions

Despite the challenges, the field of epigenetic liquid biopsy for neurometabolic disorders is rapidly advancing, with several promising future directions.

- Integration with Artificial Intelligence (AI) and Machine Learning: AI and machine learning algorithms are pivotal for processing complex cfDNA methylation data, improving diagnostic accuracy, and identifying novel methylation signatures [7]. AI can enhance biomarker discovery, optimize classification models, and aid in the interpretation of complex genomic and epigenomic landscapes [91].
- Multi-Omics Approaches: Combining cfDNA methylation profiling with other omics data, such as proteomics (e.g., neurofilament light chain, tau, α-synuclein), transcriptomics (cfRNA), and metabolomics, can provide a more comprehensive and holistic understanding of disease mechanisms and progression [90]. This integrated approach can capture multi-layered genomic and epigenomic information, enhancing diagnostic and prognostic precision [78].
- Longitudinal Studies and Biomarker Validation: Large-scale, prospective longitudinal studies are essential to validate the diagnostic and prognostic utility of CSF cfDNA methylation biomarkers across diverse patient populations and disease stages [18]. Such studies will help establish robust reference ranges, track dynamic changes over time, and correlate molecular findings with clinical outcomes and treatment responses.
- Development of Less Invasive CSF Collection Methods: Research into less invasive or alternative methods for accessing CNS-derived biomarkers, potentially through advanced blood-based approaches that can overcome BBB limitations or novel micro-invasive techniques, could broaden the applicability of these powerful molecular tools.
- Therapeutic Monitoring and Precision Medicine: Given the reversible nature of epigenetic modifications, cfDNA methylation biomarkers hold significant potential for monitoring the efficacy of epigenetic therapies or other targeted interventions in real-time [11]. This aligns with the principles of precision medicine, allowing for tailored therapeutic strategies based on an individual’s unique molecular profile and disease response [4].

## 9. Conclusions

Epigenetic profiling of cell-free DNA in cerebrospinal fluid represents a burgeoning frontier in the diagnosis and management of metabolic brain diseases. These disorders, characterized by their clinical heterogeneity, nonspecific early symptoms, and the limitations of traditional diagnostic methods, present a significant unmet clinical need. The ability of CSF-derived cfDNA to provide a direct, molecular-level window into CNS pathology, circumventing the blood–brain barrier and offering insights into specific brain cell-type injury, inflammatory signaling, and metabolic reprogramming, positions it as a highly promising source of biomarkers.

Future Research Directions:

- Systematic benchmarking of cfDNA epigenetic profiling techniques (e.g., cfMeDIP-seq, EM-seq, bisulfite sequencing) in CSF to determine sensitivity, reproducibility, and cell-type resolution across platforms.
- Longitudinal studies in preclinical models and patient cohorts to map cfDNA methylation dynamics in response to CNS metabolic stress or therapeutic interventions.
- Development of computational frameworks for integrative deconvolution of cfDNA sources, enabling refined tissue- and cell-type attribution within complex CNS pathologies.

Considerations for Integration into Clinical Practice:

- Standardization of protocols for cfDNA extraction, library preparation, and data analysis to enable cross-study comparability and clinical scalability.
- Integration of cfDNA epigenetic profiles with other omics modalities (e.g., proteomics, metabolomics) to create comprehensive disease signatures.
- Development of minimally invasive CSF collection techniques or surrogate biomarkers to enhance patient accessibility and clinical feasibility.

Current methodologies, including cfMeDIP-seq and targeted bisulfite sequencing, are continually evolving to address the challenges of ultra-low-input cfDNA, with newer enzymatic approaches, such as EM-seq, demonstrating improved efficiency and reduced DNA degradation. The inherent tissue-specificity of DNA methylation patterns allows for precise deconvolution of cfDNA origin, offering an unparalleled resolution for understanding localized brain dysfunction. This molecular granularity provides a significant advantage over conventional CSF markers and neuroimaging, enabling potentially earlier and more accurate diagnosis, dynamic monitoring of disease progression, and assessment of therapeutic response.

While technical hurdles related to DNA concentration, assay standardization, and complex bioinformatics remain, ongoing advancements in AI-driven analysis and multi-omics integration are poised to overcome these limitations. The ethical and logistical considerations associated with CSF collection, alongside the cost-effectiveness of these advanced assays, require careful consideration for broader clinical implementation. Nevertheless, there is strong potential of epigenetic cfDNA analysis in CSF for providing non-invasive, molecular-level insights into CNS metabolic. Continued research and collaborative efforts are essential to translate this promising technology into routine clinical practice, ultimately improving diagnostic timelines, guiding personalized therapies, and enhancing outcomes for patients afflicted with metabolic brain diseases.

## Figures and Tables

**Table 2:** Comparison of DNA methylation sequencing technologies, highlighting their sensitivity, cost, resolution, input requirements, and suitability for cerebrospinal-fluid cfDNA analysis.

Method	Sensitivity/Coverage	Relative Cost	Resolution	Min DNA Input
cfMeDIP-seq	Moderate genome-wide signal; efficient enrichment of methylated CpGs; good fragment capture in cfDNA3	Moderate	Regional (~100–200 bp)	~10 ng
Targeted Bisulfite-Seq	High in chosen loci; poor coverage elsewhere due to bisulfite damage1	Low–Moderate	Base-pair	5–10 ng
RRBS	Enriched CpG islands, lower genome coverage; high damage limits input3	Low	Base-pair	~10 ng
WGBS	Gold-standard complete methylome; high-depth increases costs	High	Base-pair	≥100 ng
EM-seq	High conversion accuracy, minimal degradation with low input4	Moderate–High	Base-pair	~100 pg
EPIC Array	Quantitative at defined CpGs only	Low	Probe-level (CpG sites)	≥200 ng
TAPS	Minimal DNA damage; preserves fragment integrity; ≥10 ng cfDNA usage in plasma cfDNA5	Moderate	Base-pair	~10–20 ng
UBS-seq	~13× faster bisulfite reaction; lower DNA damage and background noise in cfDNAs6	Moderate	Base-pair	1–100 cells (~1–10 ng)
LABS	Linear amplification preserves rare cfDNA fragments; single-base genome-wide data from <10 pg7	Moderate–High	Base-pair	<10 pg (~sub-nanogram)
Nanopore Methylation	Single-molecule; long reads; still modest per-site accuracy (~90–95%)8	Moderate	Base-pair and long-range	~10 ng

**Table 3:** Comparative overview—imaging vs. CSF biomarkers [100,101,102,103,104,105,106,107,108,109,110].

Modality	Measured Target	Quantitative/Qualitative Output	Diagnostic Accuracy	Limitations
CSF Lactate [100,101,102,103,104]	Anaerobic CNS metabolism	4.4 mmol/L in MELAS vs. ~1.6 in controls; cut-off > 2.2 mmol/L; AUC 0.994	High sensitivity (94–100%) and specificity (100%) in pediatric series	Requires lumbar puncture; affected by seizures/infection
1H-MRS (MELAS) [105]	Brain lactate peak (lactate/Cr)	0.40 ± 0.05 (frontal), 0.32 ± 0.03 (occipital) vs. zero controls; correlated with CSF lactate (r = 0.85)	Qualitative, binary presence detection	Not quantitative; low dynamic range
31P-MRS (Muscle MELAS) [105]	Pi/PCr ratio	Elevated post-exercise; lowered post-recovery	Reflects peripheral mitochondrial dysfunction	Indirect CNS measure; muscle-specific
MRI (MELAS) [105]	Stroke-like lesions	FLAIR/T2 hyperintensities, inverted lactate double peak, NAA/Cr low (~0.79 vs. 1.8–2.2)	Visual identification of lesions	Structural, post-lesion; no early detection
PET/CSF in Alzheimer’s	Aβ + tau PET vs. CSF Aβ42/t-tau	PET AUC 0.92–0.93; CSF Aβ42/t-tau AUC 0.93–0.94; Sens 97%, Spec 83%	Equally accurate; CSF slightly better	Both require LP or PET; cost
GLUT1 Deficiency Syndrome	CSF glucose and CSF/serum ratio	CSF glucose: 34–44% of blood; CSF/serum ratio ~0.34–0.44; no standardized lactate cut-off but often low-normal.	FDG-PET: Lenticular/thalami SUV ratio cut-off ≥ 1.54 discriminates GLUT1-DS vs. epilepsy controls with 100% sensitivity and 98% specificity. Widespread hypometabolism in thalamus, cerebellum, and temporal cortex by SPM	CSF glucose remains a more direct, earlier marker; PET shows high accuracy but requires high-cost imaging

**Table 4:** Proposed CSF cfDNA epigenetic biomarkers in metabolic brain diseases. Epigenome-wide profiling in CSF shows distinct CNS-derived methylation patterns in genes linked to neuronal development and energy metabolism.

Disease	Proposed cfDNA Epigenetic Features	Potential Advantages
MELAS [37,111,112,113]	Neuron/glia-specific methylation shifts affecting mitochondrial genes	Early detection of cell-type-specific injury; more precise than lactate
Lysosomal Disorders (e.g., Niemann-Pick, Gaucher) [114]	Methylation alterations in microglia/neuron cfDNA tied to inflammation and lipid pathways	Better detection of neuroinflammation vs. unstable small molecules
GLUT1DS [35,89,111,115,116]	cfDNA methylation changes in glucose transport/metabolism genes (e.g., SLC2A1 targets)	Detects CNS metabolic stress even if glucose is near-normal; complements new metabolic markers
Neurodegenerative-Metabolic (e.g., PD, AD, MS) [28,38,100]	cfDNA profiles reflecting insulin signaling, oxidative stress, and synaptic integrity	Enables differentiation from pure neurodegeneration

## Data Availability

No data used in the study.

## References

[1] Bais S., Kumari R., Dongre N., Panwar A.S. (2024). Insights into neurometabolic diseases. AIMS Mol. Sci..

[2] Fernández-Eulate G., Carreau C., Benoist J., Lamari F., Rucheton B., Shor N., Nadjar Y. (2022). Diagnostic approach in adult-onset neurometabolic diseases. J. Neurol. Neurosurg. Psychiatry.

[3] Otsuji R., Fujioka Y., Hata N., Kuga D., Hatae R., Sangatsuda Y., Nakamizo A., Mizoguchi M., Yoshimoto K. (2024). Liquid Biopsy for Glioma Using Cell-Free DNA in Cerebrospinal Fluid. Cancers.

[4] CD Genomics (2023). Cell-free DNA as a Biomarker in Precision Oncology. https://www.cd-genomics.com/resource-data-precision-oncology-cfdna.html.

[5] Cornelli L., Van Paemel R., Ferro Dos Santos M.R., Roelandt S., Willems L., Vandersteene J., Baert E., Mus L.M., Van Roy N., De Wilde B. (2024). Diagnosis of pediatric central nervous system tumors using methylation profiling of cfDNA from cerebrospinal fluid. Clin. Epigenetics.

[6] BioIVT Unpacking the FDA Draft Guidance on Biopsies in Clinical Trials. https://bioivt.com/blogs/unpacking-the-fda-draft-guidance-on-biopsies-in-clinical-trials.

[7] Akabane M., Imaoka Y., Kawashima J., Pawlik T.M. (2025). Advancing precision medicine in hepatocellular carcinoma: Current challenges and future directions in liquid biopsy, immune microenvironment, single nucleotide polymorphisms, and conversion therapy. Hepatic Oncol..

[8] Armakolas A., Kotsari M., Koskinas J. (2023). Liquid Biopsies, Novel Approaches and Future Directions. Cancers.

[9] Aydın Ş., Özdemir S., Adıgüzel A. (2025). The Potential of cfDNA as Biomarker: Opportunities and Challenges for Neurodegenerative Diseases. J. Mol. Neurosci..

[10] Life in the Lab Stuff (2025). Cell-Free DNA (cfDNA) vs. Circulating Tumor DNA (ctDNA) Explained. https://www.thermofisher.com/blog/life-in-the-lab/cfdna-vs-ctdna/.

[11] Gaitsch H., Franklin R.J.M., Reich D.S. (2023). Cell-free DNA-based liquid biopsies in neurology. Brain.

[12] Liu F., Su Y., Liu X., Zhao L., Wu Z., Liu Y., Zhang L. (2025). Cell-free DNA: A metabolic byproduct with diagnostic and prognostic potential in rheumatic disorders. Front. Pharmacol..

[13] Pathak A., Tomar S., Pathak S. (2023). Epigenetics and Cancer: A Comprehensive Review. Asian Pac. J. Cancer Biol..

[14] Al Aboud N.M., Tupper C., Jialal I. (2025). Genetics, Epigenetic Mechanism. StatPearls.

[15] Lehmann-Werman R., Neiman D., Zemmour H., Moss J., Magenheim J., Vaknin-Dembinsky A., Rubertsson S., Nellgård B., Blennow K., Zetterberg H. (2016). Identification of tissue-specific cell death using methylation patterns of circulating DNA. Proc. Natl. Acad. Sci. USA.

[16] Jin Y., Allen E.G., Jin P. (2022). Cell-free DNA methylation as a potential biomarker in brain disorders. Epigenomics.

[17] Cai M., Zhou J., McKennan C., Wang J. (2024). scMD facilitates cell type deconvolution using single-cell DNA methylation references. Commun. Biol..

[18] McEwen A.E., Leary S.E.S., Lockwood C.M. (2020). Beyond the Blood: CSF-Derived cfDNA for Diagnosis and Characterization of CNS Tumors. Front. Cell Dev. Biol..

[19] Zhang J., Wang W., Li Y. (2023). A characteristic comparison of cell-free DNA isolated from plasma and cerebrospinal fluid in patients with cancer. J. Clin. Oncol..

[20] (2024). File:EPIC-seq Workflow.png. Wikimedia Commons. https://commons.wikimedia.org/w/index.php?title=File:EPIC-seq_workflow.png&oldid=859065986.

[21] Pia S., Lui F. (2025). Melas Syndrome. StatPearls.

[22] File:Mitochondrial Inheritance.svg. Wikimedia Commons. Published 8 March 2022. https://commons.wikimedia.org/w/index.php?title=File:Mitochondrial_inheritance.svg&oldid=636413970.

[23] (2024). File:Mitochondrial DNA lg.jpg. Wikimedia Commons. https://commons.wikimedia.org/w/index.php?title=File:Mitochondrial_DNA_lg.jpg&oldid=946178322.

[24] MedlinePlus (2013). Mitochondrial Encephalomyopathy, Lactic Acidosis, and Stroke-like Episodes: MedlinePlus Genetics. https://medlineplus.gov/genetics/condition/mitochondrial-encephalomyopathy-lactic-acidosis-and-stroke-like-episodes/.

[25] El-Hattab A.W., Almannai M., Scaglia F., Adam M.P., Feldman J., Mirzaa G.M. (2001). MELAS. GeneReviews^®^.

[26] (2023). File:Modified Gomori Trichrome Stain Showing Several Ragged Red Fibers.jpg. Wikimedia Commons. https://commons.wikimedia.org/w/index.php?title=File:Modified_Gomori_trichrome_stain_showing_several_ragged_red_fibers.jpg&oldid=763868130.

[27] Yu J., Paterson C., Davies P., Palmer J.C., Higgins J.P.T., Kurian K.M. (2025). Evaluating liquid biopsy biomarkers for early detection of brain metastasis: A systematic review. Neuro-Oncol. Pract..

[28] Liguori C., Stefani A., Fernandes M., Cerroni R., Mercuri N.B., Pierantozzi M. (2022). Biomarkers of Cerebral Glucose Metabolism and Neurodegeneration in Parkinson’s Disease: A Cerebrospinal Fluid-Based Study. J. Parkinsons Dis..

[29] BMJ Best Practice (2025). Common Hereditary Lysosomal Storage Diseases—Symptoms, Diagnosis and Treatment|BMJ Best Practice US. https://bestpractice.bmj.com/topics/en-us/1021.

[30] Sun A. (2017). Lysosomal storage disease overview. Ann. Transl. Med..

[31] Stirnemann J., Belmatoug N., Camou F., Serratrice C., Froissart R., Caillaud C., Levade T., Astudillo L., Serratrice J., Brassier A. (2017). A Review of Gaucher Disease Pathophysiology, Clinical Presentation and Treatments. Int. J. Mol. Sci..

[32] Hughes D., Mikosch P., Belmatoug N., Carubbi F., Cox T., Goker-Alpan O., Kindmark A., Mistry P., Poll L., Weinreb N. (2019). Gaucher Disease in Bone: From Pathophysiology to Practice. J. Bone Miner. Res..

[33] Patterson M., Adam M.P., Feldman J., Mirzaa G.M. (2000). Niemann-Pick Disease Type C. GeneReviews^®^.

[34] De Vivo D. (2024). Glucose Transporter Type 1 Deficiency Syndrome—NORD (National Organization for Rare Disorders). https://rarediseases.org/rare-diseases/glucose-transporter-type-1-deficiency-syndrome/.

[35] Peters T.M.A., Merx J., Kooijman P.C., Noga M., de Boer S., van Gemert L.A., Salden G., Engelke U.F., Lefeber D.J., van Outersterp R.E. (2022). Novel CSF biomarkers of GLUT1 deficiency syndrome: Implications beyond the brain’s energy deficit. medRxiv.

[36] Steele G., Klepper J., Poduri A. (2021). GLUT1 Deficiency Syndrome (SLC2A1). Epilepsy Foundation. https://www.epilepsy.com/causes/metabolic/glut1.

[37] Mattox A.K., Yan H., Bettegowda C. (2019). The potential of cerebrospinal fluid-based liquid biopsy approaches in CNS tumors. Neuro-Oncol..

[38] Bonomi C.G., De Lucia V., Mascolo A.P., Assogna M., Motta C., Scaricamazza E., Sallustio F., Mercuri N.B., Koch G., Martorana A. (2021). Brain energy metabolism and neurodegeneration: Hints from CSF lactate levels in dementias. Neurobiol. Aging.

[39] (2024). File:Gaucher Disease—Very High Mag.jpg. Wikimedia Commons. https://commons.wikimedia.org/w/index.php?title=File:Gaucher_disease_-_very_high_mag.jpg&oldid=890184076.

[40] (2022). File:Epigenetic Mechanisms.jpg. Wikimedia Commons. https://commons.wikimedia.org/w/index.php?title=File:Epigenetic_mechanisms.jpg&oldid=721049823.

[41] Yu X., He H., Wen J., Xu X., Ruan Z., Hu R., Wang F., Ju H. (2025). Diabetes-related cognitive impairment: Mechanisms, symptoms, and treatments. Open Med..

[42] Ma K., Xiao Y. (2024). Mechanisms of Cognitive Decline in Newly Diagnosed Diabetics: A Review of Pathophysiological Contributions and Intervention Strategies. MEDS Basic Med..

[43] Zhang S., Zhang Y., Wen Z., Yang Y., Bu T., Bu X., Ni Q. (2023). Cognitive dysfunction in diabetes: Abnormal glucose metabolic regulation in the brain. Front. Endocrinol..

[44] Duarte J.M. (2015). Metabolic Alterations Associated to Brain Dysfunction in Diabetes. Aging Dis..

[45] Abbatecola A.M., Arosio B., Cerasuolo M., Auriemma M.C., Di Meo I., Langiano E., Rizzo M.R. (2024). Common neurodegenerative pathways in brain aging, cognitive decline, type 2 diabetes & metabolic syndrome. J. Gerontol. Geriatr..

[46] Mayo Clinic Staff (2024). Mild Cognitive Impairment—Symptoms and Causes. https://www.mayoclinic.org/diseases-conditions/mild-cognitive-impairment/symptoms-causes/syc-20354578.

[47] Summers K. (2025). Study Unlocks How Diabetes Distorts Memory and Reward Processing. UNLV. https://www.unlv.edu/news/release/study-unlocks-how-diabetes-distorts-memory-and-reward-processing.

[48] Bhasin G., Flores E., Crew L.A., Wirt R.A., Ortiz A.A., Kinney J.W., Hyman J.M. (2025). ACC reward location information is carried by hippocampal theta synchrony and suppressed in a Type 2 Diabetes model. J. Neurosci..

[49] (2024). File:CSF Circulation.png. Wikimedia Commons. https://commons.wikimedia.org/w/index.php?title=File:CSF_circulation.png&oldid=859369762.

[50] MedlinePlus (2025). Cerebrospinal Fluid (CSF) Analysis: MedlinePlus Lab Test Information. https://medlineplus.gov/lab-tests/cerebrospinal-fluid-csf-analysis/.

[51] Czarniak N., Kamińska J., Matowicka-Karna J., Koper-Lenkiewicz O.M. (2023). Cerebrospinal Fluid–Basic Concepts Review. Biomedicines.

[52] (2024). File:CtDNA in circulation.png. Wikimedia Commons. https://commons.wikimedia.org/w/index.php?title=File:CtDNA_in_circulation.png&oldid=871699736.

[53] (2023). File:Blausen 0617 LumbarPuncture.png. Wikimedia Commons. https://commons.wikimedia.org/w/index.php?title=File:Blausen_0617_LumbarPuncture.png&oldid=825516397.

[54] (2024). File:DNAme landscape.png. Wikimedia Commons. https://commons.wikimedia.org/w/index.php?title=File:DNAme_landscape.png&oldid=873650162.

[55] Lossi L., Castagna C., Merighi A. (2024). An Overview of the Epigenetic Modifications in the Brain under Normal and Pathological Conditions. Int. J. Mol. Sci..

[56] Younesian S., Yousefi A.M., Momeny M., Ghaffari S.H., Bashash D. (2022). The DNA Methylation in Neurological Diseases. Cells.

[57] Lio C.W.J., Rao A. (2019). TET Enzymes and 5hmC in Adaptive and Innate Immune Systems. Front. Immunol..

[58] Williams K., Christensen J., Helin K. (2011). DNA methylation: TET proteins—Guardians of CpG islands?. EMBO Rep..

[59] Locke W.J., Guanzon D., Ma C., Liew Y.J., Duesing K.R., Fung K.Y., Ross J.P. (2019). DNA Methylation Cancer Biomarkers: Translation to the Clinic. Front. Genet..

[60] Miller J.L., Grant P.A. (2013). The role of DNA methylation and histone modifications in transcriptional regulation in humans. Epigenet. Dev. Dis..

[61] Hiemenz M., Chang J. (2025). DNA Methylation to the Rescue: An Update for Pathologists Navigating the Future of Precision Tumor Diagnostics. College of American Pathologists. https://www.cap.org/member-resources/articles/dna-methylation-to-the-rescue-an-update-for-pathologists-navigating-the-future-of-precision-tumor-diagnostics.

[62] Kakodkar P., Conway K., Santana-Santos L., McCord M., Sukhanova M., Castellani R., Jamshidi P. (2025). DNA methylation-based classification of the central nervous system tumors, achievements, and challenges. Advanced Concepts and Strategies in Central Nervous System Tumors.

[63] Tang W., Wan S., Yang Z., Teschendorff A.E., Zou Q. (2018). Tumor origin detection with tissue-specific miRNA and DNA methylation markers. Bioinformatics.

[64] Chen K., Li Z., Kirsh B.O., Luo P., Pedersen S., Bucur R.C., Rukavina N.A., Bruce J.P., Danesh A., Riverin M. (2024). Plasma cell-free DNA methylomes for hepatocellular carcinoma detection and monitoring after liver resection or transplantation. medRxiv.

[65] Wilson S.L., Shen S.Y., Harmon L., Burgener J.M., Triche T., Bratman S.V., De Carvalho D.D., Hoffman M.M. (2021). Sensitive and reproducible cell-free methylome quantification with synthetic spike-in controls. bioRxiv.

[66] Biorad Epigenetics and Chromatin Structure|Bio-Rad. https://www.bio-rad.com/en-us/applications-technologies/epigenetics-chromatin-structure?ID=LUSNMFHYP.

[67] CD Genomics (2023). Innovation in WGBS: New Methods for DNA Methylation Analysis. https://www.cd-genomics.com/epigenetics/resource-new-wgbs-technology-pbat-wgbs.html.

[68] (2023). File:Bisulfite-Reaction.png. Wikimedia Commons. https://commons.wikimedia.org/w/index.php?title=File:Bisulfite-reaction.png&oldid=824921164.

[69] (2012). File:Wiki Bisulfite Sequencing Figure 1 Small.png. Wikipedia. https://en.wikipedia.org/wiki/File:Wiki_Bisulfite_sequencing_Figure_1_small.png.

[70] (2017). Abcam. Bisulfite Sequencing in DNA Methylation Analysis|Abcam. https://www.abcam.com/en-us/knowledge-center/dna-and-rna/bisulfite-sequencing.

[71] De Koker A., Raman L., Van Paemel R., Van der Linden M., Van de Velde S., Van der Leest P., Van Gaever B., Zaka A., Vermaelen K., Demedts I. (2025). Development of circulating cell-free DNA reduced representation bisulfite sequencing for clinical methylomics diagnostics. bioRxiv.

[72] (2024). What Are the Limitations of Reduced Representation Bisulfite Sequencing (RRBS)?|AAT Bioquest. https://www.aatbio.com/resources/faq-frequently-asked-questions/what-are-the-limitations-of-reduced-representation-bisulfite-sequencing-rrbs.

[73] CD Genomics (2021). DNA Methylation Array Workflow & Analysis: Process Optimization and Data Insights. https://www.cd-genomics.com/epigenetics/resource-dna-methylation-array-workflow-optimization-analysis.html.

[74] CD Genomics (2021). Overview of EM-seq: A New Detection Approach for DNA Methylation—CD Genomics. https://www.cd-genomics.com/epigenetics/resource-dna-methylation-detection-em-seq.html.

[75] Illumina (2019). Infinium MethylationEPIC Kit|Methylation Profiling Array for EWAS. https://www.illumina.com/products/by-type/microarray-kits/infinium-methylation-epic.html.

[76] Sebastian A., Silvy M., Coiffard B., Reynaud-Gaubert M., Magdinier F., Chiaroni J., Picard C., Pedini P. (2024). A review of cell-free DNA and epigenetics for non-invasive diagnosis in solid organ transplantation. Front. Transplant..

[77] Tan W.Y., Nagabhyrava S., Ang-Olson O., Das P., Ladel L., Sailo B., He L., Sharma A., Ahuja N. (2024). Translation of Epigenetics in Cell-Free DNA Liquid Biopsy Technology and Precision Oncology. Curr. Issues Mol. Biol..

[78] Pollard C.A., Saito E.R., Burns J.M., Hill J.T., Jenkins T.G. (2024). Considering Biomarkers of Neurodegeneration in Alzheimer’s Disease: The Potential of Circulating Cell-Free DNA in Precision Neurology. J. Pers. Med..

[79] Southwood D., Singh S., Chatterton Z. (2022). Brain-derived cell-free DNA. Neural Regen. Res..

[80] Jin Y., Conneely K.N., Ma W., Naviaux R.K., Siddique T., Allen E.G., Guingrich S., Pascuzzi R.M., Jin P. (2025). Whole-genome bisulfite sequencing of cell-free DNA unveils age-dependent and ALS-associated methylation alterations. Cell Biosci..

[81] Rados M., Klotz S., Regelsberger G., Stögmann E., Landegger L.D., Fetahu I.S. (2025). Distinct cerebrospinal fluid DNA methylation signatures linked to Alzheimer’s disease. bioRxiv.

[82] Giannini L.A.A., Boers R.G., van der Ende E.L., Poos J.M., Jiskoot L.C., Boers J.B., van IJcken W.F.J., Dopper E.G., Pijnenburg Y.A.L., Seelaar H. (2024). Distinctive cell-free DNA methylation characterizes presymptomatic genetic frontotemporal dementia. Ann. Clin. Transl. Neurol..

[83] Ma Y., Wang W., Liu S., Qiao X., Xing Y., Zhou Q., Zhang Z. (2023). Epigenetic Regulation of Neuroinflammation in Alzheimer’s Disease. Cells.

[84] Bai I., Keyser C., Zhang Z., Rosolia B., Hwang J.-Y., Zukin R.S., Yan J. (2024). Epigenetic regulation of autophagy in neuroinflammation and synaptic plasticity. Front. Immunol..

[85] (2024). File:Blausen 0870 TypesofNeuroglia.png. Wikimedia Commons. https://commons.wikimedia.org/w/index.php?title=File:Blausen_0870_TypesofNeuroglia.png&oldid=859366013.

[86] Cologna S.M., Cluzeau C.V., Yanjanin N.M., Blank P.S., Dail M.K., Siebel S., Toth C.L., Wassif C.A., Lieberman A.P., Porter F.D. (2014). Human and mouse neuroinflammation markers in Niemann-Pick disease, type C1. J. Inherit. Metab. Dis..

[87] Boddupalli C.S., Nair S., Belinsky G., Gans J., Teeple E., Nguyen T.H., Mehta S., Guo L., Kramer M.L., Ruan J. (2022). Neuroinflammation in neuronopathic Gaucher disease: Role of microglia and NK cells, biomarkers, and response to substrate reduction therapy. elife.

[88] Fawal M.A., Davy A. (2018). Impact of Metabolic Pathways and Epigenetics on Neural Stem Cells. Epigenet. Insights.

[89] Peters T.M.A., Merx J., Kooijman P.C., Noga M., de Boer S., van Gemert L.A., Salden G., Engelke U.F.H., Lefeber D.J., van Outersterp R.E. (2023). Novel cerebrospinal fluid biomarkers of glucose transporter type 1 deficiency syndrome: Implications beyond the brain’s energy deficit. J. Inherit. Metab. Dis..

[90] Laufer B.I., Hasegawa Y., Zhang Z., Hogrefe C.E., Del Rosso L.A., Haapanen L., Hwang H., Bauman M.D., Van de Water J., Taha A.Y. (2022). Multi-omic brain and behavioral correlates of cell-free fetal DNA methylation in macaque maternal obesity models. Nat. Commun..

[91] Myrou A., Barmpagiannos K., Ioakimidou A., Savopoulos C. (2025). Molecular Biomarkers in Neurological Diseases: Advances in Diagnosis and Prognosis. Int. J. Mol. Sci..

[92] Wang Y., Li J., Li J., Yang S., Huang Y., Liu X., Fan Y., King I., Li Y., Li Y. (2025). cfDecon: Accurate and Interpretable methylation-based cell type deconvolution for cell-free DNA. bioRxiv.

[93] Zuccato J.A., Patil V., Mansouri S., Voisin M., Chakravarthy A., Shen S.Y., Nassiri F., Mikolajewicz N., Trifoi M., Skakodub A. (2023). Cerebrospinal fluid methylome-based liquid biopsies for accurate malignant brain neoplasm classification. Neuro-Oncol..

[94] Riviere-Cazaux C., Dong X., Mo W., Kumar R., Dai C., Carlstrom P.L., Munoz-Casabella A., Ghadimi K., Nesvick L.C., Andersen M.K. (2025). Longitudinal Glioma Monitoring via Cerebrospinal Fluid Cell-Free DNA. Clin. Cancer Res..

[95] Huang J., Wang L. (2019). Cell-Free DNA Methylation Profiling Analysis-Technologies and Bioinformatics. Cancers.

[96] Iser F., Hinz F., Hoffmann D.C., Grassl N., Güngoör C., Meyer J., Dörner L., Hofmann L., Kelbch V., Göbel K. (2024). Cerebrospinal Fluid cfDNA Sequencing for Classification of Central Nervous System Glioma. Clin. Cancer Res..

[97] Hu R., Li S., Stackpole M.L., Li Q., Zhou X.J., Li W. (2025). cfTools: An R/Bioconductor package for deconvolving cell-free DNA via methylation analysis. Bioinform. Adv..

[98] Keukeleire P., Makrodimitris S., Reinders M. (2023). Cell type deconvolution of methylated cell-free DNA at the resolution of individual reads. NAR Genom. Bioinform..

[99] Research and Markets (2025). Liquid Biopsy Market Forecast to Reach US$13.44 Billion by 2030 at 12.19% CAGR|Next-Gen Sequencing and PCR Breakthroughs Fuel Innovation in Liquid Biopsy Diagnostics. GlobeNewswire News Room. https://www.globenewswire.com/news-release/2025/04/09/3058183/28124/en/Liquid-Biopsy-Market-Forecast-to-Reach-US-13-44-Billion-by-2030-at-12-19-CAGR-Next-Gen-Sequencing-and-PCR-Breakthroughs-Fuel-Innovation-in-Liquid-Biopsy-Diagnostics.html.

[100] Albanese M., Zagaglia S., Landi D., Boffa L., Nicoletti C.G., Marciani M.G., Mandolesi G., Marfia G.A., Buttari F., Mori F. (2016). Cerebrospinal fluid lactate is associated with multiple sclerosis disease progression. J. Neuroinflamm..

[101] Magner M., Szentiványi K., Svandová I., Ješina P., Tesařová M., Honzík T., Zeman J. (2011). Elevated CSF-lactate is a reliable marker of mitochondrial disorders in children even after brief seizures. Eur. J. Paediatr. Neurol..

[102] Abe K., Fujimura H., Nishikawa Y., Yorifuji S., Mezaki T., Hirono N., Nishitani N., Kameyama M. (1991). Marked reduction in CSF lactate and pyruvate levels after CoQ therapy in a patient with mitochondrial myopathy, encephalopathy, lactic acidosis and stroke-like episodes (MELAS). Acta Neurol. Scand..

[103] Xu S., Jiang J., Chang L., Zhang B., Zhu X., Niu F. (2024). Multisystem clinicopathologic and genetic analysis of MELAS. Orphanet J. Rare Dis..

[104] Yamada K., Toribe Y., Yanagihara K., Mano T., Akagi M., Suzuki Y. (2012). Diagnostic accuracy of blood and CSF lactate in identifying children with mitochondrial diseases affecting the central nervous system. Brain Dev..

[105] Guerrero-Molina M.P., Bernabeu-Sanz Á., Ramos-González A., Morales-Conejo M., Delmiro A., Domínguez-González C., Arenas J., Martín M.A., González de la Aleja J. (2024). Magnetic resonance spectroscopy in MELAS syndrome: Correlation with CSF and plasma metabolite levels and change after glutamine supplementation. Neuroradiology.

[106] Natsume J., Ishihara N., Azuma Y., Nakata T., Takeuchi T., Tanaka M., Sakaguchi Y., Okai Y., Ito Y., Yamamoto H. (2021). Lenticular nuclei to thalamic ratio on PET is useful for diagnosis of GLUT1 deficiency syndrome. Brain Dev..

[107] Marin-Valencia I., Kocabas A., Rodriguez-Navas C., Miloushev V.Z., González-Rodríguez M., Lees H., Henry K.E., Vaynshteyn J., Longo V., Deh K. (2024). Imaging brain glucose metabolism in vivo reveals propionate as a major anaplerotic substrate in pyruvate dehydrogenase deficiency. Cell Metab..

[108] Meyer P.T., Frings L., Rücker G., Hellwig S. (2017). 18F-FDG PET in Parkinsonism: Differential Diagnosis and Cognitive Impairment in Parkinson’s disease. J. Nucl. Med..

[109] Probasco J.C., Solnes L., Nalluri A., Cohen J., Jones K.M., Zan E., Javadi M.S., Venkatesan A. (2017). Abnormal brain metabolism on FDG-PET/CT is a common early finding in autoimmune encephalitis. Neurol. Neuroimmunol. Neuroinflamm..

[110] Akman C.I., Provenzano F., Wang D., Engelstad K., Hinton V., Yu J., Tikofsky R., Ichese M., De Vivo D.C. (2015). Topography of brain glucose hypometabolism and epileptic network in glucose transporter 1 deficiency. Epilepsy Res..

[111] Cappuccio G., Pinelli M., Alagia M., Donti T., Day-Salvatore D.L., Veggiotti P., De Giorgis V., Lunghi S., Vari M.S., Striano P. (2017). Biochemical phenotyping unravels novel metabolic abnormalities and potential biomarkers associated with treatment of GLUT1 deficiency with ketogenic diet. PLoS ONE.

[112] Pan L., McClain L., Shaw P., Donnellan N., Chu T., Finegold D., Peters D. (2020). Non-invasive epigenomic molecular phenotyping of the human brain via liquid biopsy of cerebrospinal fluid and next generation sequencing. Eur. J. Neurosci..

[113] Yang C., Pan R.Y., Guan F., Yuan Z. (2024). Lactate metabolism in neurodegenerative diseases. Neural Regen. Res..

[114] Otto C., Kalantzis R., Kübler-Weller D., Kühn A.A., Böld T., Regler A., Strathmeyer S., Wittmann J., Ruprecht K., Heelemann S. (2024). Comprehensive analysis of the cerebrospinal fluid and serum metabolome in neurological diseases. J. Neuroinflamm..

[115] Mastrangelo M., Manti F., Ricciardi G., Cinnante E.M.C., Cameli N., Beatrice A., Tolve M., Pisani F. (2024). The diagnostic and prognostic role of cerebrospinal fluid biomarkers in glucose transporter 1 deficiency: A systematic review. Eur. J. Pediatr..

[116] Leen W.G., Wevers R.A., Kamsteeg E.J., Scheffer H., Verbeek M.M., Willemsen M.A. (2013). Cerebrospinal fluid analysis in the workup of GLUT1 deficiency syndrome: A systematic review. JAMA Neurol..

